# Field-Resolved Three-Phase Dephosphorisation in Molten Steel: Euler–Euler–DPM Modelling of Bottom-Blown Oxygen–Lime-Powder Injection

**DOI:** 10.3390/ma19173715

**Published:** 2026-08-31

**Authors:** Hongyang Wang, Wenxuan Mo, Kai Dong

**Affiliations:** 1 Beijing Zhiruzhenyi Superconducting Technology Co., Ltd., Beijing 102200, China; 15916787989@163.com; 2 Institute for Carbon Neutrality, University of Science and Technology Beijing, Beijing 100083, China; 3 Jiangxi Province Key Laboratory of Power Batteries Energy Storage Materials, Jiangxi University of Science and Technology, Ganzhou 341000, China

**Keywords:** molten-steel dephosphorisation, gas–slag–metal reactions, Euler–Euler–DPM modelling, bottom-blown oxygen–CaO powder injection

## Abstract

**Featured Application:**

**Abstract:**

Dephosphorisation in oxygen steelmaking depends on more than the equilibrium phosphorus partition ratio. It also depends on where gas, slag, metal and injected lime powder coexist while the bath is stirred. We develop a gas–slag–metal–particle reaction model for bottom-blown oxygen–CaO powder injection by coupling Euler–Euler transport of liquid steel, mixed slag, and gas with a discrete phase model (DPM) for CaO particles. The local source terms include oxygen dissolution, FeO/Fe_2_O_3_ conversion, CO/CO_2_ buffering, competitive C/Si/P oxidation, $P_{2}O_{5}$ formation, $C_{2}S$–$C_{3}P$ fixation, reaction heat, and phase-wise mass conservation. Bubble swarms, dispersed slag, and emulsified metal–slag contact are represented through mean-field interfacial area densities tied to local phase fractions and mixing. Two simulated composition states have the same initial phosphorus content but different C, Si, and dissolved O levels; they are therefore compared as Case H and Case L rather than as a carbon-only test. Under the selected closures, Case H shows stronger decarburisation and CO-supported plume motion, whereas Case L retains more ${\mathrm{FeO}}_{x}$ and dissolved oxygen near the slag–metal interface. In both states, calculated P removal is confined mainly to locations where ${\mathrm{FeO}}_{x}$ supply, CaO availability, $P_{2}O_{5}$ generation, and $C_{2}S$–$C_{3}P$ fixation overlap. These observations are conditional on the reported parameters, a production mesh accompanied only by a two-grid qualitative sensitivity check, one time step, and the early transient considered here. Quantitative validation, systematic grid/time-step studies, closure-sensitivity tests, and controlled-composition simulations are required before the framework is used for process prediction.

## 1. Introduction

Phosphorus control remains one of the strictest constraints in oxygen steelmaking. Carbon, silicon, manganese, sulphur, and inclusions all affect cleanliness and downstream processing, but phosphorus is especially difficult because it remains soluble in molten iron and is not removed efficiently in the reducing blast-furnace environment. Its removal is therefore shifted to oxidative steelmaking or hot-metal pretreatment, where phosphorus must be transferred into a highly basic oxidising slag [1,2,3]. Excess phosphorus increases cold brittleness, reduces toughness, and narrows the processing window for low-phosphorus steels. As low-phosphorus and ultra-low-phosphorus products become more demanding, dephosphorisation has to be treated as a coupled process involving slag volume, lime consumption, iron loss, injection practice, temperature schedule, and the low-carbon smelting route [3,4,5].

The equilibrium conditions for phosphorus removal are comparatively well understood. Classical work linked the phosphorus distribution ratio to oxygen potential, basicity, temperature, and iron-oxide content, leading to empirical and semi-thermodynamic descriptions based on phosphorus partitioning, optical basicity, phosphorus capacity, and multicomponent slag activities [2,6,7,8,9]. These studies point to a consistent requirement: high CaO activity, sufficient FeO oxygen-supply capacity, lower temperature, and low $P_{2}O_{5}$ activity favour transfer from metal to slag. Once a $2CaO\cdot SiO_{2}$–$3CaO\cdot P_{2}O_{5}$ solid solution forms, phosphorus can be fixed and the risk of rephosphorisation decreases [10,11,12,13,14,15]. The CALculation of PHAse Diagrams (CALPHAD) approach and FactSage descriptions now cover major steelmaking-slag subsystems, including CaO–SiO_2_–${\mathrm{FeO}}_{x}$–$P_{2}O_{5}$–MgO–Al_2_$O_{3}$–MnO, and can calculate multiphase equilibria among liquid slag, solid phases, and gas [16,17,18,19]. The larger uncertainty is not whether phosphorus removal is thermodynamically allowed, but where that capacity is realised in a moving converter bath.

Kinetic studies have clarified many rate-controlling steps, but most of them come from laboratory systems or semi-empirical reaction models. Lime dissolution, FeO oxygen supply, metal–slag mass transfer, solid phosphate formation, slag viscosity, and stirring intensity have all been examined as controls on the apparent dephosphorisation rate [10,11,12,13,14,15,20,21,22]. Those systems usually have clearer interface areas, controlled slag-to-metal ratios, more uniform temperatures, and smaller flow scales than an industrial converter. In an operating bath, bubble swarms, slag droplets, metal droplets, foamed slag, undissolved lime, FeO-rich pockets, and temperature gradients coexist. The interfacial area is renewed by the flow, and a local oxygen potential cannot be replaced by a furnace-average value. For such dispersed interfaces, Euler–Euler multifluid modelling is useful because it represents mutually penetrating continua through phase-volume averaging and can carry gas holdup, slag holdup, interphase momentum exchange, and volumetric source terms at the furnace scale [23,24,25,26]. Laboratory kinetic constants therefore need to be reclosed by local flow, phase distribution, and interface renewal before they can be used as industrial reaction rates.

Industrial dephosphorisation studies often focus on flow optimisation and endpoint correlations. Bottom blowing, top–bottom combined blowing, powder injection, foamed-slag control, and low-alkalinity multi-stage smelting are usually evaluated through mixing time, bubble dispersion, powder penetration, liquid-level fluctuation, or improved slag–metal contact [3,27,28]. Conventional flow simulations describe velocity, gas holdup, free-surface motion, and particle trajectories, but they do not directly answer the metallurgical question: where is [P] consumed, through which gas–slag–metal pathway, and at what local rate is it oxidised and transferred into slag? A flow field alone does not contain the chemical closures needed for phosphorus partitioning, $FeO/Fe_{2}O_{3}$ oxygen potential, $CO/CO_{2}$ gas buffering, lime dissolution, $C_{2}S$–$C_{3}P$ fixation, and competitive oxidation of C, Si, and P [1,2,3,16,29]. If these reactions are added only as furnace-average post-processing estimates, the local dephosphorisation path remains hidden.

Effective-equilibrium reaction-zone models provide a second route between thermodynamics and process prediction. They assume that a local reaction zone reaches, or approaches, equilibrium and then embed thermodynamic calculations into a process model through empirical mass-transfer coefficients, mixing parameters, and reaction-zone volume fractions. This approach can describe composition evolution in ladle refining, weld-pool reactions, and steelmaking reaction-path calculations [19,30]. For dephosphorisation, the advantage is clear: mature thermodynamic databases can be coupled with interfacial balance and bulk mass transfer. The limitation is also clear: the position, volume, and interface area of the reaction zone usually have to be prescribed or calibrated, so the model does not directly recover how macroscopic flow controls local [P] consumption, $P_{2}O_{5}$ generation, and solid phosphate formation. Such models are useful for rapid process prediction, but they cannot by themselves locate the active reaction zones in a strongly coupled gas–slag–metal flow.

Here, we build a gas–slag–metal three-phase reaction framework for dephosphorisation in molten steel. The Euler–Euler multifluid formulation describes the spatial occupation and interphase exchange of gas, continuous mixed slag, and metal. A discrete phase model (DPM) tracks lime-powder’s entry, residence, and capture by phase regions. Local conservative source terms describe $O_{2}$ dissolution, FeO generation and oxygen release, $CO/CO_{2}$ oxygen-potential buffering, C/Si/P competitive oxidation, phosphorus transfer to slag, $P_{2}O_{5}$ generation, and $C_{2}S$–$C_{3}P$ fixation. The model does not attempt to resolve every bubble surface. Instead, the contact areas of bubble swarms, slag droplets, and metal–slag emulsions are written as mean-field interfacial area densities at the control-volume scale, allowing rates to vary with local phase fraction, mixing, and particle contact [23,25,26,31]. This structure brings thermodynamic targets, kinetic limits, flow renewal, and interphase mass feedback into the same conservative equation set.

Bottom-blown oxygen–lime-powder injection is used as the test case because it contains the essential couplings: gas-phase oxygen supply, powder dissolution, slag–metal reaction, bubble stirring, iron-oxide generation, decarburisation, and solid phosphate fixation. Bottom-blown oxygen–lime or limestone-powder processes have drawn attention because they may accelerate slag formation, improve lime utilisation, strengthen bath mixing, support low-phosphorus steelmaking, and reduce energy use or slag volume [3,27,28]. Existing work, however, still tends to emphasise injection schedules, powder transport, stirring, and endpoint indicators. A field-resolved reaction description is needed to show how powder entry changes local CaO activity, FeO oxygen-supply capacity, the metal [P] consumption path, and the location of $P_{2}O_{5}$ fixation [1,15,29]. This case therefore tests whether gas–slag, gas–metal, and slag–metal interfacial reactions can be represented together.

The framework keeps the simplifications required for an industrial-scale calculation. Unresolved bubbles and fragmented interfaces are represented by mean-field interfacial area densities rather than by single-bubble direct numerical simulation. Slag activity, solid precipitation, and lime dissolution are closed with process-oriented relations that still require calibration against experimental or plant data. Temperature, turbulent mixing, foamed-slag morphology, and particle-size distribution can all change the local rate. The present model is therefore used to examine the time- and space-dependent tendency of [P] transfer while maintaining consistency among phase region, oxygen potential, and reactant inventory. Its fields should be read as conditional model outputs and mechanistic hypotheses, not as quantitatively validated plant predictions. A calculation that reports metal [P], slag $P_{2}O_{5}$, CaO consumption, $FeO/Fe_{2}O_{3}$ feedback, and gas-phase $CO/CO_{2}$ evolution can nevertheless show interactions that are hidden by furnace-average estimates.

## 2. Method

The method embeds oxygen bottom blowing, liquid-steel oxidation, slag-oxide reactions, CaO-particle contact, reversible dephosphorisation, and reaction heat release in a locally closed gas–slag–metal network. The gas phase supplies $O_{2}$ and buffers oxygen potential through $CO/CO_{2}$. The metal phase sets the competitive oxidation order through the activities of *C*, Si, *P*, and dissolved oxygen *O*. The slag phase responds through $FeO/Fe_{2}O_{3}$ redox conversion, liquid-CaO capacity, phosphorus partitioning, and the virtual solid-fixation capacity of $2CaO\cdot SiO_{2}$–$3CaO\cdot P_{2}O_{5}$. Low-oxygen or high-$P_{2}O_{5}$ zones allow rephosphorisation, whereas high-dissolved-oxygen zones allow Fe+[O]→FeO to continue supplying iron oxide to the slag. The slag is not split into additional fluid phases. Liquid slag, free CaO, and the $C_{2}S$–$C_{3}P$ fixation phase are treated as convertible compositions inside the same continuous slag phase. This choice avoids extra fluid phases in the three-phase mixing zone while retaining the main chemical effects of CaO-assisted melting, phosphate fixation, and iron-oxide oxygen-potential adjustment. In the Euler–Euler formulation, all reaction sources must preserve phase ownership, mass conservation, boundedness, and consistency with locally available reactants [23,24,25].

### 2.1. Phase Definition Conservation Equations

We use coupled Euler–Euler–DPM modelling. The Eulerian field contains three interpenetrating continua: liquid steel/metal, continuous mixed slag, and gas. The metal phase contains *C*, Si, *P*, *O*, and balance Fe; the slag phase contains CaO, $SiO_{2}$, FeO, $Fe_{2}O_{3}$, $P_{2}O_{5}$, free CaO, the virtual $C_{2}S$–$C_{3}P$ fixation phase, and an inert remainder; and the gas phase contains $O_{2}$, CO, $CO_{2}$, and Ar. Each chemical species is transported in its own host phase. Diagnostic reaction variables are used only for post-processing and do not enter compositional conservation. The DPM tracks lime particles through the Eulerian field. When a particle enters an effective slag phase or an FeO-rich reaction film, its calculated mass loss is added once to the slag-phase CaO/free-CaO inventory in the next flow-field update.

The reported calculations use the Euler–Euler formulation. The volume of fluid (VOF) method is mentioned only as an alternative for systems with a small number of resolved free interfaces; it is not used to generate the results in this paper. Here, the target is the averaged reactive contact generated by many bubbles, slag droplets, emulsified metal–slag regions, and undissolved particles within an industrial control volume. Resolving each bubble or slag drop with VOF would make the cost grow with bubble number and breakup while leaving furnace-scale dispersed reaction sources difficult to stabilise. The Euler–Euler method instead solves phase volume fractions, phase velocities, and phase-owned species for interpenetrating continua, while the DPM retains individual powder trajectories [23,24,25,26,31,32].

The three-phase volume fraction is closed as

(1) $$\alpha_{g}+\alpha_{s}+\alpha_{m}=1$$

In Equation (1), $\alpha_{g}$, $\alpha_{s}$, and $\alpha_{m}$ are the gas-, slag-, and metal-phase volume fractions, respectively; free CaO and $C_{2}S$–$C_{3}P$ are internal components of the slag phase and are not used as independent fluid phases.

When molten steel is the primary phase, its volume fraction is back-calculated from the secondary-phase volume fractions:

(2) $$\alpha_{m}=1-\alpha_{s}-\alpha_{g}$$

Equation (2) ensures that errors in the secondary-phase solution do not violate the three-phase volume closure.

The mass conservation of any phase *q* is written as

(3) $$\frac{\partial (\alpha_{q}\rho_{q})}{\partial t}+\nabla \cdot \left(\alpha_{q}\rho_{q}u_{q}\right)=S_{q}$$

In Equation (3), $\rho_{q}$ and $u_{q}$ are the density and velocity of phase *q*, respectively, and $S_{q}$ is the phase mass source. In this model, $S_{g}$ comes from changes in $O_{2}$, CO, and $CO_{2}$; $S_{s}$ comes from oxide generation and CaO inventory input; and $S_{m}$ follows from metal-element consumption and iron-balance conservation.

The conservation of momentum for phase *q* is written as

(4) $$\frac{\partial (\alpha_{q}\rho_{q}u_{q})}{\partial t}+\nabla \cdot \left(\alpha_{q}\rho_{q}u_{q}u_{q}\right)=-\alpha_{q}\nabla p+\nabla \cdot \left(\alpha_{q}\tau_{q}\right)+\alpha_{q}\rho_{q}g+\sum_{p\neq q}M_{pq}$$

In Equation (4), *p* is the shared pressure field, $\tau_{q}$ is the corresponding stress tensor, and $M_{pq}$ is the interphase momentum exchange. This relation expresses the basic characteristics of the Euler–Euler model: the phases share space but may have different velocities and are coupled through interphase momentum sources.

The Reynolds-averaged flow uses the *k*–ω shear-stress transport (SST) turbulence model. The SST equations, phase properties, Schiller–Naumann interphase drag, and Ranz–Marshall heat-transfer closure used in the archived case are given in Appendix B.

The transport of the explicit component *i* within the phase follows a conserved form:

(5) $$\frac{\partial (\alpha_{q}\rho_{q}Y_{i,q})}{\partial t}+\nabla \cdot \left(\alpha_{q}\rho_{q}u_{q}Y_{i,q}\right)=\nabla \cdot \left(\alpha_{q}\rho_{q}D_{i,q}^{\mathrm{eff}}\nabla Y_{i,q}\right)+S_{i,q}$$

In Equation (5), $Y_{i,q}$ is the mass fraction of component *i* in phase *q*, $D_{i,q}^{\mathrm{eff}}$ is the effective diffusion coefficient, and $S_{i,q}$ is the mass source of the component. Each source term is added only within the phase to which the component belongs, thereby avoiding incorrect cross-phase diffusion of *C*, Si, *P*, and *O* in the metal or of oxides in the slag.

The particle position and momentum equations are reported in Appendix A.2.3. In the archived setup, the Eulerian field drives particle momentum and sensible heat, whereas native DPM feedback of momentum, turbulence, and sensible heat to the Eulerian phases is disabled. The only particle-to-continuous-phase feedback is the custom, conservative CaO mass source produced by particle dissolution.

The equivalent contact area of phases *i* and *j* in a control volume is

(6) $$A_{ij}^{cell}=a_{ij}V_{c}$$

In Equation (6), $V_{c}$ is the control volume, and $a_{ij}$ is the mean-field interfacial area density. The gas–metal, gas–slag, and metal–slag reactions are not integrated bubble by bubble from individual surfaces; their volume-averaged sources depend on local phase fractions and characteristic dispersion scales.

The continuous phase weights adopt a bounded volume fraction function:

(7) $$\omega_{q}=\min\left(1,\max\left(0,\frac{\alpha_{q}}{\alpha_{\mathrm{ref}}}\right)\right)$$

In Equation (7), $\alpha_{\mathrm{ref}}=5.0\times 10^{-2}$. This weight causes the dispersed-bubble contact area to change mainly with the gas-phase content, while requiring the contacted continuous phase to reach a resolvable local occupation level.

The gas–metal interface area density is closed as

(8) $$a_{gm}=2.0\;\frac{6\alpha_{g}\omega_{m}}{d_{gm}}I_{gm}$$

In Equation (8), $d_{gm}=2.5\times 10^{-3}\;m$, and the coefficient 2.0 represents enhancement of the gas–metal contact area in the bottom-blown plume. The gas–metal reaction is active only when both $\alpha_{g}$ and $\alpha_{m}$ are at least $10^{-8}$, for which $I_{gm}=1$; otherwise it is closed.

The gas–slag interface area density is closed as

(9) $$a_{gs}=0.15\;\frac{6\alpha_{g}\omega_{s}}{d_{gs}}I_{gs}$$

In Equation (9), $d_{gs}=5.0\times 10^{-3}\;m$, and the coefficient 0.15 weakens the gas–slag redox channel relative to gas–metal oxygen supply. The same $10^{-8}$ volume-fraction contact threshold is used for $I_{gs}$.

The metal–slag interface area density is closed as

(10) $$a_{ms}=\frac{6\alpha_{m}\alpha_{s}}{d_{ms}}I_{ms}$$

In Equation (10), $d_{ms}=2.0\times 10^{-2}\;m$. To prevent trace numerical phase fractions from activating the slag–metal closure, $I_{ms}=1$ only when $\alpha_{m}\geq 0.01$ and $\alpha_{s}\geq 0.01$.

The volumetric source term of an interfacial reaction *r* is obtained by multiplying the interfacial flux by the corresponding area density.

(11) $$R_{r}^{\prime \prime \prime }=a_{ij}J_{r}$$

In Equation (11), $J_{r}$ is the finite rate flux per unit interface area. Mixed interface reactions are therefore subject to two-level constraints: first, effective volume fractions of both phases must exist simultaneously; and second, the reactant inventory limits described later must be satisfied.

The native phase-species equations use the constant-dilute molecular diffusivity stored in the ANSYS Fluent 2026 R1 (ANSYS, Inc., Canonsburg, PA, USA) case together with the solver’s turbulent-diffusion closure. For the reported calculations, $D_{i,q}=2.88\times 10^{-5}\;m^{2}\;s^{-1}$ for all three Eulerian mixtures, $Sc_{t}=0.70$, and no additional user-defined diffusion floor is applied ($D_{\mathrm{floor}}=0$). The selected $Sc_{t}$ is the Fluent default for turbulent species transport and is an empirical closure of order unity, rather than a measured steel–slag material property [33,34].

(12) $$D_{i,q}^{\mathrm{eff}}=D_{i,q}+\frac{\nu_{t}}{Sc_{t}}+D_{\mathrm{floor}}$$

In Equation (12), $D_{i,q}$ is the molecular diffusivity, $\nu_{t}$ is the turbulent kinematic viscosity, $Sc_{t}$ is the turbulent Schmidt number, and $D_{\mathrm{floor}}$ denotes any separately imposed numerical diffusivity. The production UDF contains neither DEFINE_DIFFUSIVITY nor a custom floor, so $D_{\mathrm{floor}}=0$ in the reported implementation. Appendix B lists the associated species-transport switches. Because scalar flux data are unavailable for this multiphase steelmaking system, $Sc_{t}=0.70$ was retained as the documented solver default; it should be varied in a transport-sensitivity study before quantitative prediction.

### 2.2. Closed Reaction Network

The reaction network is organised around four physical channels: gas-phase oxygen supply and $CO/CO_{2}$ buffering; competitive oxidation of *C*, Si, and *P* in the metal; $FeO/Fe_{2}O_{3}$ redox in the slag; and fixation of $P_{2}O_{5}$ after CaO dissolution. Bottom-blown oxygen–CaO powder is not a single slag–metal equilibrium problem. Direct oxygen supply, FeO-film formation, decarburisation-bubble generation, preferential silicon oxidation, and CaO-particle fluxing all occur near the nozzle [35,36,37,38,39,40,41]. High-temperature experiments and process models show that the apparent phosphorus-removal rate changes with FeO oxygen supply, interface renewal by metal droplets or bubbles, CaO dissolution, and solid phosphate precipitation. Each forward and reverse channel is therefore written as a locally limited source term rather than as a single furnace-averaged dephosphorisation rate [42,43,44,45,46,47,48].

To avoid repeating elementary formulae in both text and table, the reaction set is reported once in Table 1. The table includes the gas–metal formation of FeO, direct oxygen dissolution, FeO oxygen release, CO afterburning, ${\mathrm{CO}}_{2}$ cracking, C/Si/P oxidation, limited rephosphorisation, ${\mathrm{FeO/Fe}}_{2}O_{3}$ gas–slag redox, free-CaO exchange, and $C_{2}S$–$C_{3}P$ fixation. This compact form keeps the stoichiometric closure visible while avoiding a long sequence of one-line equations. The individual reaction choices follow standard FeO and oxygen-potential thermodynamics, dilute-solution C/O and Si/O equilibria, dephosphorisation partitioning, lime dissolution, and phosphate-fixation studies [2,13,15,20,49,50,51,52].

Table 1 maps each rate to its closure reaction and to the corresponding gas, metal, and slag inventory changes. It defines the mass-conservation logic of the reaction network: gas–metal reactions change gas $O_{2}/CO/CO_{2}$ and metal dissolved oxygen, metal–slag reactions transfer Si and *P* oxidation products into slag, and gas–slag reactions change $CO/CO_{2}$ while adjusting the $FeO/Fe_{2}O_{3}$ ratio. These channels share the same local gas oxygen potential, metal dissolved-oxygen pool, and slag-oxide inventory.

Table 1 reports only one forward and one reverse phosphorus reaction. In the UDF, the forward kinetic coefficient contains a contact-factor contribution and an interfacial-area contribution. These internal terms describe how the unresolved metal–slag contact enters the same overall rate; they are not two independently completed phosphorus reactions and are never attached to separate species-source hooks. The $L_{P}$ relation first supplies the forward or reverse driving force. The resulting kinetic candidate is then bounded by the same local P inventory and by the oxygen allocated after competition with C oxidation, Si oxidation, and FeO formation. Only the limited signed net rate enters the conserved P, O, and $P_{2}O_{5}$ sources, as detailed in Appendix A.2.1. The earlier presentation of the two internal terms as separate rows obscured this serial control and has therefore been corrected without changing the implemented coefficients or source values.

Gas–slag reactions change gas and slag inventories at the same time. For example, $2FeO+CO_{2}\to Fe_{2}O_{3}+CO$ consumes gas-phase $CO_{2}$, generates gas-phase CO, lowers slag FeO, and raises slag $Fe_{2}O_{3}$. The resulting $CO/CO_{2}$ ratio then feeds back into the $FeO/Fe_{2}O_{3}$ target ratio and the metal-side oxidation capacity through the oxygen-potential relation. Conversely, CO generated by decarburisation can be oxidised by $O_{2}$ to $CO_{2}$, reducing the $O_{2}$ available for FeO formation and metal oxygen supply. The three-phase reaction network is therefore closed within each control volume rather than arranged as a one-way sequence of phase-interface reactions.

### 2.3. Computability Constraints

Several numerical limits are imposed to keep the explicit source update conservative and bounded. All inventory limits use an effective time step with a lower bound of $10^{-9}\;s$ so that a very small or undefined time scale cannot produce singular source terms. Thermodynamic and kinetic functions use the local energy-equation temperature when it is valid; otherwise, they fall back to the bath temperature of 1873K. The effective temperature is clipped to 1200–2300K, which keeps the linearised thermodynamic expressions, Arrhenius rates, and reaction enthalpies inside the intended steelmaking range [2,49].

Component and phase source terms are bounded by the locally available species mass, local phase inventory, and a global volumetric mass-source cap. These limits prevent negative mass fractions, phase exhaustion, and isolated reaction-film spikes without changing the stoichiometry listed in Table 1. CaO particles release material only when the effective slag contact, including real slag, FeO-rich reaction film, and newly generated FeO, exceeds 0.02 in local volume-fraction measure. A single particle substep is limited to 20% mass loss, and complete loss is applied only when the remaining particle mass is below the prescribed cutoff. Boundary deposition writes remaining CaO into the slag only when the boundary hit occurs inside an effective slag region; escape through the top gas outlet terminates particle tracking without adding CaO to the slag. These limits follow the numerical role of source-term boundedness in finite-volume multiphase calculations and the physical requirement that CaO dissolution needs local slag or FeO contact rather than pure gas-phase residence [20,53,54,55].

### 2.4. Mesh and Case Settings

**Figure 1:**
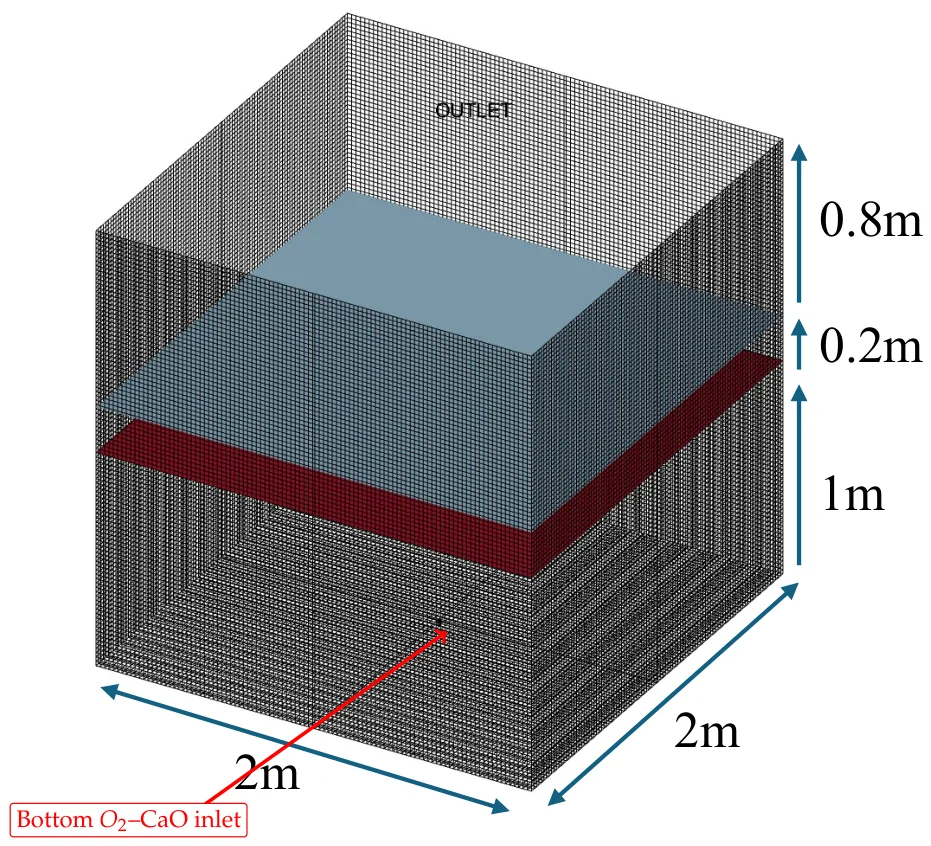
Computational domain and main mesh. The red arrow marks the central bottom boundary through which oxygen and CaO powder are injected; the top boundary is the pressure outlet.

The computational domain consists of a main fluid region, a central bottom gas–powder inlet, a top pressure outlet, and stationary walls. The mesh contains 1,020,102 hexahedral control volumes, 1,050,812 nodes, one bottom inlet surface, 10,201 pressure-outlet faces, and 50,608 wall faces. Liquid steel is the primary Eulerian phase, and continuously mixed slag and gas are secondary phases. The phases share pressure but retain separate velocities, volume fractions, and phase-owned species. Equations (8)–(10) close the gas–metal, gas–slag, and metal–slag reaction areas. Appendix B lists the remaining material, interaction, particle-injection, and solver settings. The computational domain, mesh, and bottom injection boundary are shown in Figure 1.

The gas-flow conversion, initial temperature, and three-layer phase initialisation are reported with the other calculation settings in Appendix B.

The numerical treatment first closes the reaction network and then limits the reacted amount in each time step by the local inventory. This preserves feedback among gas-phase oxygen supply, FeO oxygen supply, ${\mathrm{CO/CO}}_{2}$ buffering, CaO dissolution, phosphate fixation, and rephosphorisation, while avoiding the artificial conversion of local thermodynamic tendency into instantaneous global equilibrium.

The reported fields were obtained on the 1,020,102-cell mesh. Appendix C compares one-dimensional axial gas-composition and Eulerian phase-fraction profiles with an additional coarse mesh of approximately 132,000 cells—a reduction in cell count by a factor of about 7.7. The comparison tests whether the broad axial gas-conversion ordering and steel/gas partition persist under substantial coarsening; it does not establish three-dimensional field convergence, an observed-order grid-convergence index, or grid independence. The production time step is reported in Appendix B, and no time-step-independence claim is made.

## 3. Results and Discussion

The fields below are instantaneous outputs at the nominal analysis time t=4.0s. Case H starts from $\left(Y_{C},Y_{Si},Y_{P},Y_{O}\right)=\left(0.035,0.0010,0.0010,5.117556\times 10^{-6}\right)$, whereas Case L starts from $\left(0.001,0.0005,0.0010,1.052567\times 10^{-4}\right)$. Since C, Si, and dissolved O all differ, the comparison concerns two composition states and does not isolate the effect of carbon. The split figures use the same scales for corresponding Case H and Case L panels unless the caption states otherwise. These snapshots are used to examine reaction location and coupling at the selected time. They do not establish transient convergence or an endpoint state. The coarse-grid comparison in Appendix C shows that the broad axial gas-conversion ordering persists, while pointwise gas composition and phase fraction remain mesh-sensitive.

### 3.1. Phase Distribution and Effective Reaction Interfaces

**Figure 2:**
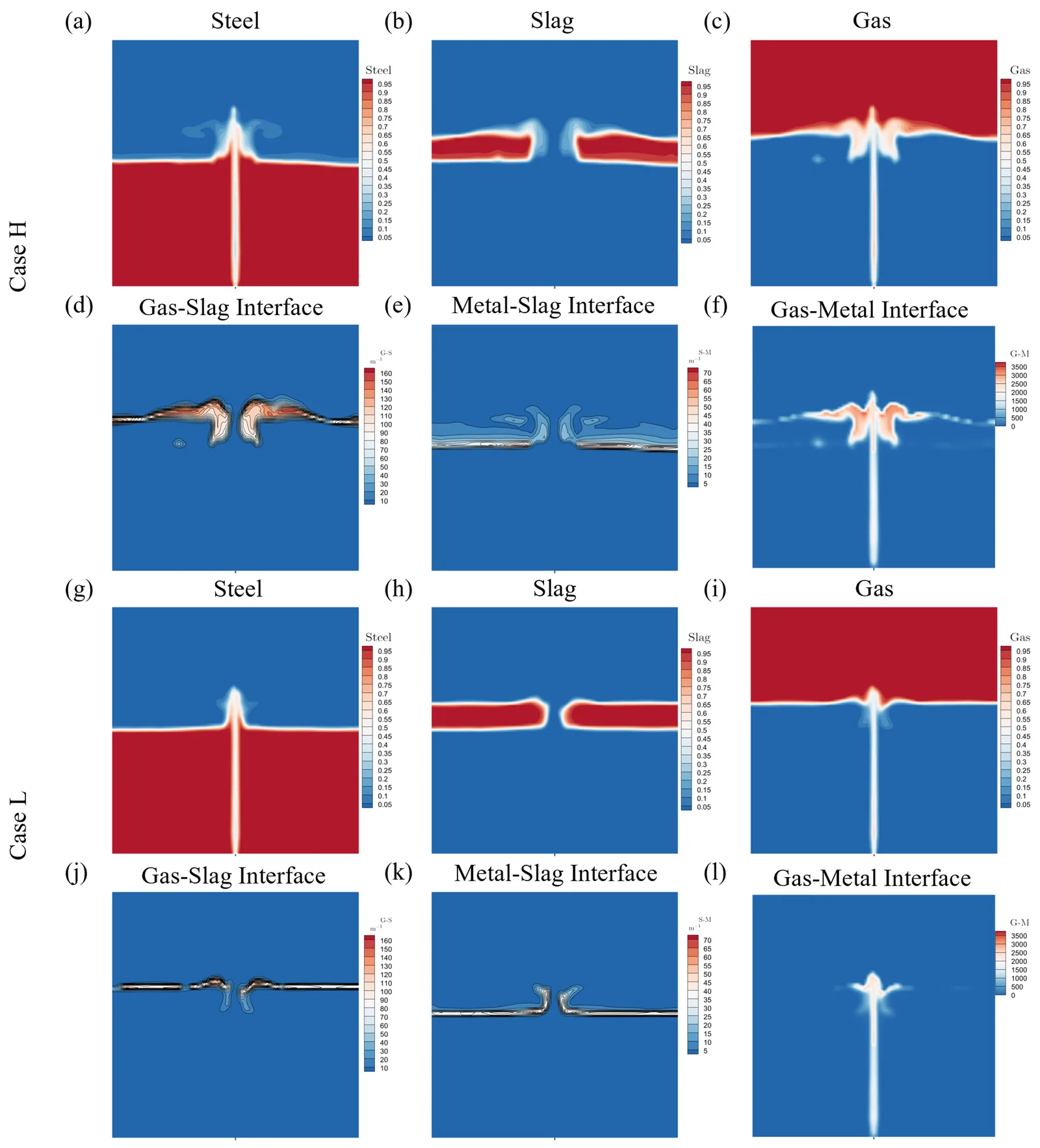
Eulerian phase distribution and mean-field interface-area densities at t=4.0s. Panels (**a**–**f**) show Case H and panels (**g**–**l**) show Case L. Within each case, the first three panels show steel, slag, and gas volume fractions, followed by gas–slag, metal–slag, and gas–metal interface-area densities. Phase volume fractions are dimensionless, and interface-area densities are in $m^{-1}$. Corresponding Case H and Case L panels use identical scales.

Figure 2 separates the phase-volume-fraction fields from the three mean-field interface-area densities. The steel and slag layers are lifted around the central plume in both cases, but the deformation is broader in Case H. The gas field in Case H also spreads farther along the slag–metal transition. Case L remains more nearly axial. The volume-fraction range of 0.05–0.95 is an Eulerian phase occupation, not a sharp geometric boundary. The interface-area closures convert this local phase overlap into reaction area per unit volume. This interpretation follows the continuum-averaged treatment of multiphase flow [23,24,25].

The largest calculated interface-area density is the gas–metal term, which reaches the order of $3500\;m^{-1}$ in the central plume and its upper branches. The gas–slag and metal–slag terms are smaller, with plotted upper scales of about 160 and $70\;m^{-1}$, respectively, and are concentrated around the disturbed upper layer. Case H produces a wider contact region, consistent with the stronger gas generation and lateral plume expansion seen below. Water-model and converter studies likewise relate bottom injection to plume expansion and bath circulation [56,57]. The calculated contact area is a carrier for source terms, however, and should not be read as a reaction rate by itself.

**Figure 3:**
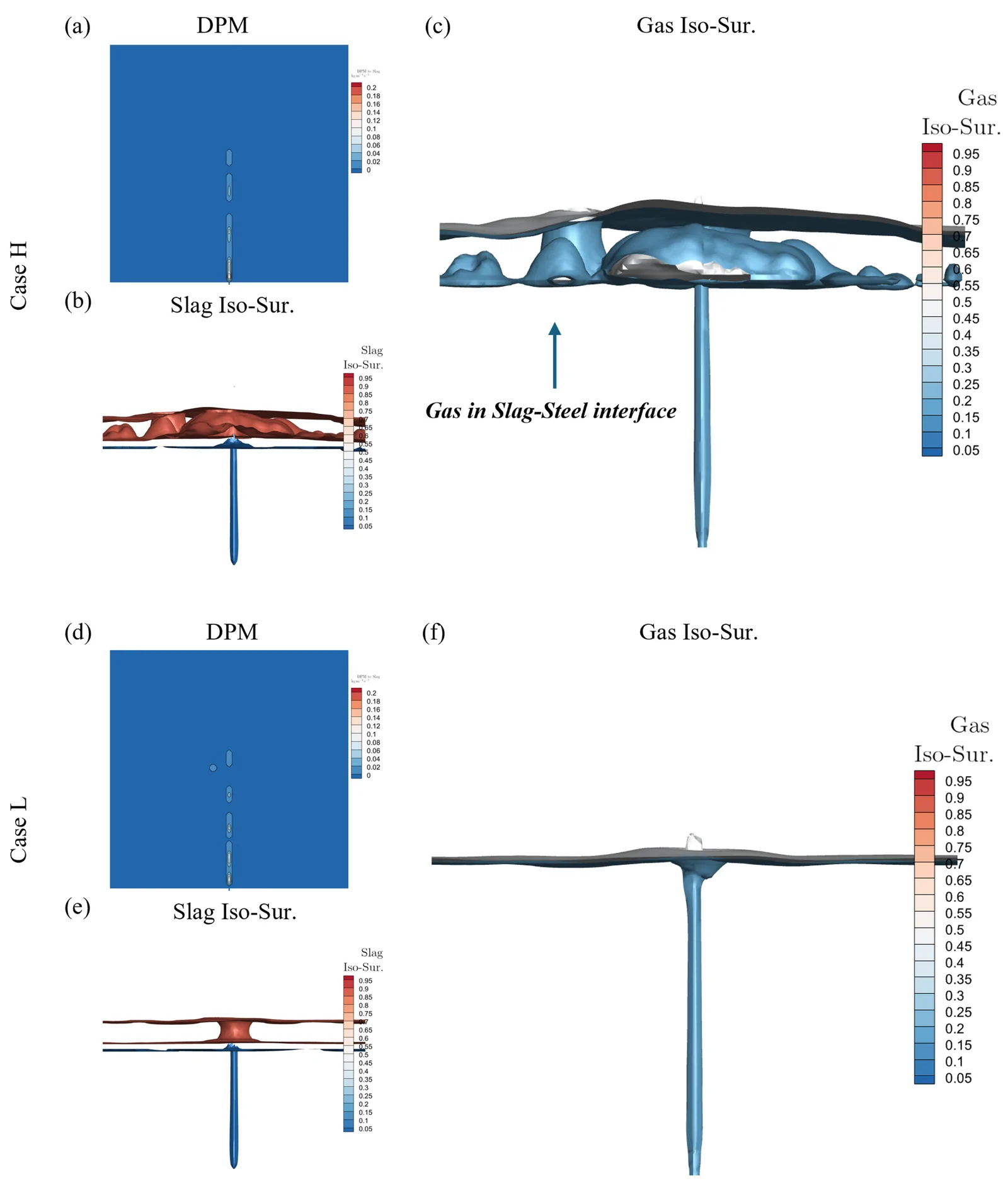
Discrete-particle CaO release and three-dimensional phase isosurfaces at t=4.0s. Panels (**a**–**c**) show Case H and panels (**d**–**f**) show Case L. Panels (**a**,**d**) show the DPM CaO-to-slag source in kg $m^{-3}s^{-1}$; panels (**b**,**e**) show slag isosurfaces and panels (**c**,**f**) show gas isosurfaces, coloured by dimensionless phase volume fraction. Corresponding Case H and Case L panels use identical scales.

Figure 3 shows the DPM CaO-to-slag source together with three-dimensional slag and gas isosurfaces. The particle source remains close to the injection axis and appears as a sequence of local release zones rather than as a uniform addition to the slag. In Case H, the gas isosurface spreads laterally after reaching the slag–metal region, and the slag isosurface is more strongly corrugated. The corresponding Case L structures are narrower. DPM is therefore retained because particle paths and local residence histories should not be replaced by a spatially uniform CaO source [32]. Powder-injection studies also show that tuyere arrangement, trajectory, and residence time affect the fraction of CaO that reaches a useful reaction environment [39,40].

The two figures describe different parts of the same closure. Figure 2 gives the mean contact area available to the Eulerian reaction network, while Figure 3 identifies where the discrete particles release CaO. A large interfacial area does not guarantee dephosphorisation if the local zone lacks ${\mathrm{FeO}}_{x}$, usable CaO, or phosphorus-fixation capacity. Furnace-scale equilibrium models can estimate partitioning but cannot locate this transient overlap [19]. Thermodynamic descriptions of slag capacity therefore need local interface and inventory information in this three-phase calculation [16,58].

### 3.2. Gas Transport and Reaction Zoning

**Figure 4:**
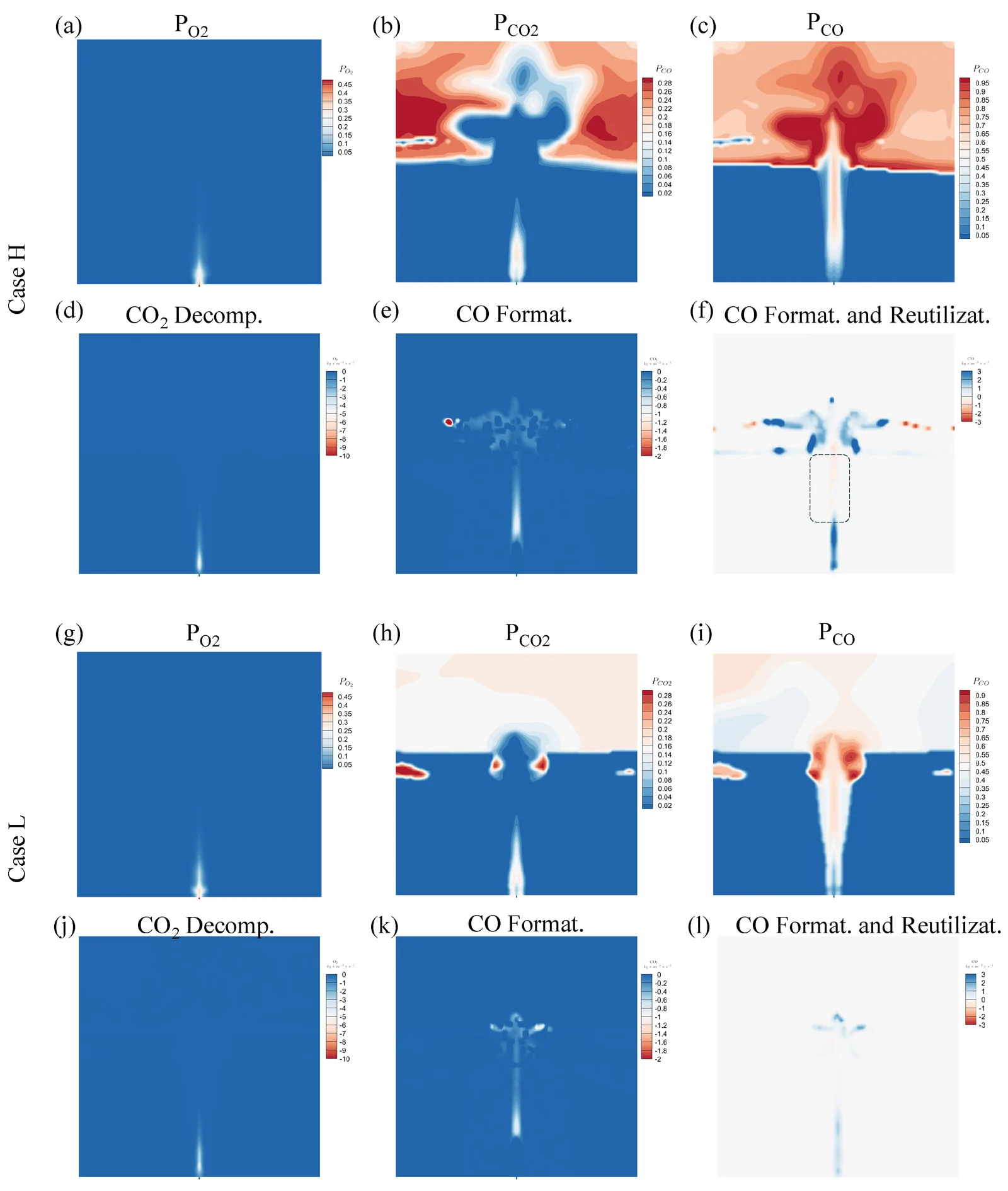
Gas partial-pressure fields and signed gas-species source terms at t=4.0s. Panels (**a**–**f**) show Case H and panels (**g**–**l**) show Case L. Panels (**a**–**c**,**g**–**i**) give $P_{O_{2}}$, $P_{CO_{2}}$, and $P_{CO}$ in atm. Panels (**d**–**f**,**j**–**l**) give ${\mathrm{CO}}_{2}$ decomposition, CO formation, and the combined CO-formation/reutilisation source in kg $m^{-3}s^{-1}$; their signs follow the plotted species-source convention. Corresponding Case H and Case L panels use identical scales.

The spatial fields in Figure 4 show that free $O_{2}$ remains confined to the lower part of the injection column in both cases. Above this region, the gas composition is dominated by CO and ${\mathrm{CO}}_{2}$. Case H develops a broad CO-rich zone around the central plume and the disturbed slag–metal layer, with local $P_{CO}$ approaching the plotted upper value of 0.95. Case L has a narrower CO distribution and more localised ${\mathrm{CO}}_{2}$ structures. Thus, the gas entering the upper bath is not simply transported inlet oxygen. It has already been modified by decarburisation, post-combustion, and gas–slag redox.

The source maps in Figure 4 locate these conversions. The oxygen-containing column supplies the lower reaction zone, CO formation follows the plume, and ${\mathrm{CO}}_{2}$ decomposition or reutilisation is concentrated near the upper mixed layer. The signs in the plotted source fields follow the species-source convention used by the UDF; the physical interpretation therefore comes from the paired species balances rather than from one colour map alone. The coupled [C]–[O]–${\mathrm{CO/CO}}_{2}$ equilibrium in liquid iron provides the basis for this buffering [51]. Carbon-bearing gas also changes slag oxygen potential through ${\mathrm{FeO}}_{x}$ reduction and reoxidation [52,59], with the free-energy relations taken from National Institute of Standards and Technology (NIST) thermochemical data [49].

**Figure 5:**
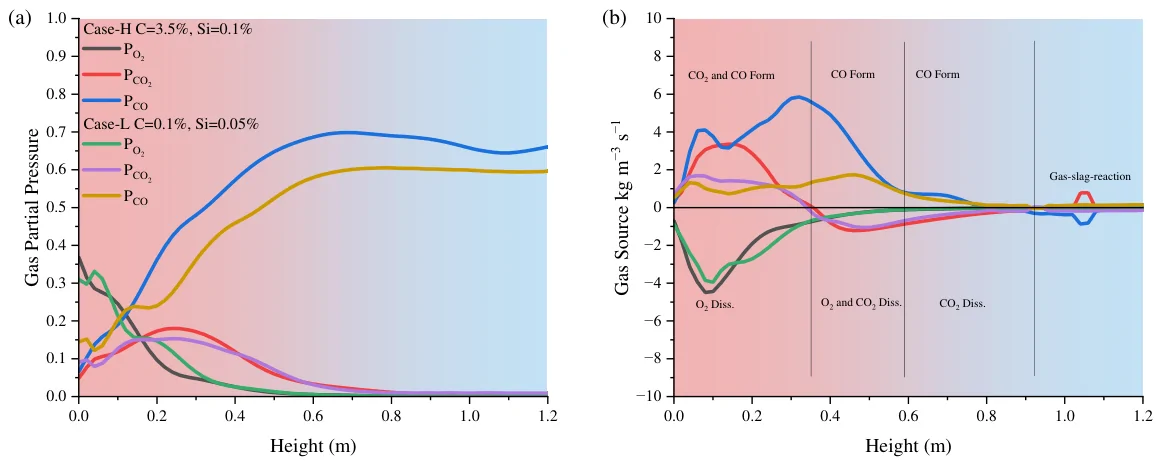
Height profiles of gas composition and reaction sources at t=4.0s. Panel (**a**) compares $P_{O_{2}}$, $P_{CO_{2}}$, and $P_{CO}$ in atm for Cases H and L. Panel (**b**) compares the corresponding signed gas-species sources in kg $m^{-3}s^{-1}$. Height is measured from the bottom inlet in m.

The height profiles in Figure 5 make the reaction zoning clearer. In Case H, $P_{O_{2}}$ falls rapidly from the inlet, while $P_{CO}$ rises through the lower and middle plume and remains high toward the upper layer. $P_{CO_{2}}$ first increases and then decays. Case L follows the same sequence but with lower CO and a more localised source distribution. The signed source profiles divide the bath into a lower oxygen-dissolution and ${\mathrm{CO/CO}}_{2}$-formation region, a middle CO-forming and gas-dissolution region, and an upper gas–slag exchange region. These zones are descriptive features of the t=4.0s field, not independently calibrated reaction-front positions.

The gas phase therefore supplies oxygen, removes carbon, maintains plume buoyancy, and redistributes ${\mathrm{FeO}}_{x}$ oxygen potential. Case H has stronger gas generation and interface renewal, whereas Case L retains more oxidising capacity near the slag–metal layer. Initial Si and dissolved O also differ, so this contrast is assigned to the full composition states. Bottom-blown oxygen–lime and lime-powder experiments similarly show that free oxygen supply alone does not determine dephosphorisation; local slag formation and CaO–FeO contact remain necessary [37,60,61]. Off-gas studies also place CO generation and post-combustion within the bath reaction balance [62].

### 3.3. CaO Supply, ${\mathrm{FeO}}_{x}$ Conversion, and Phosphorus-Bearing Slag

**Figure 6:**
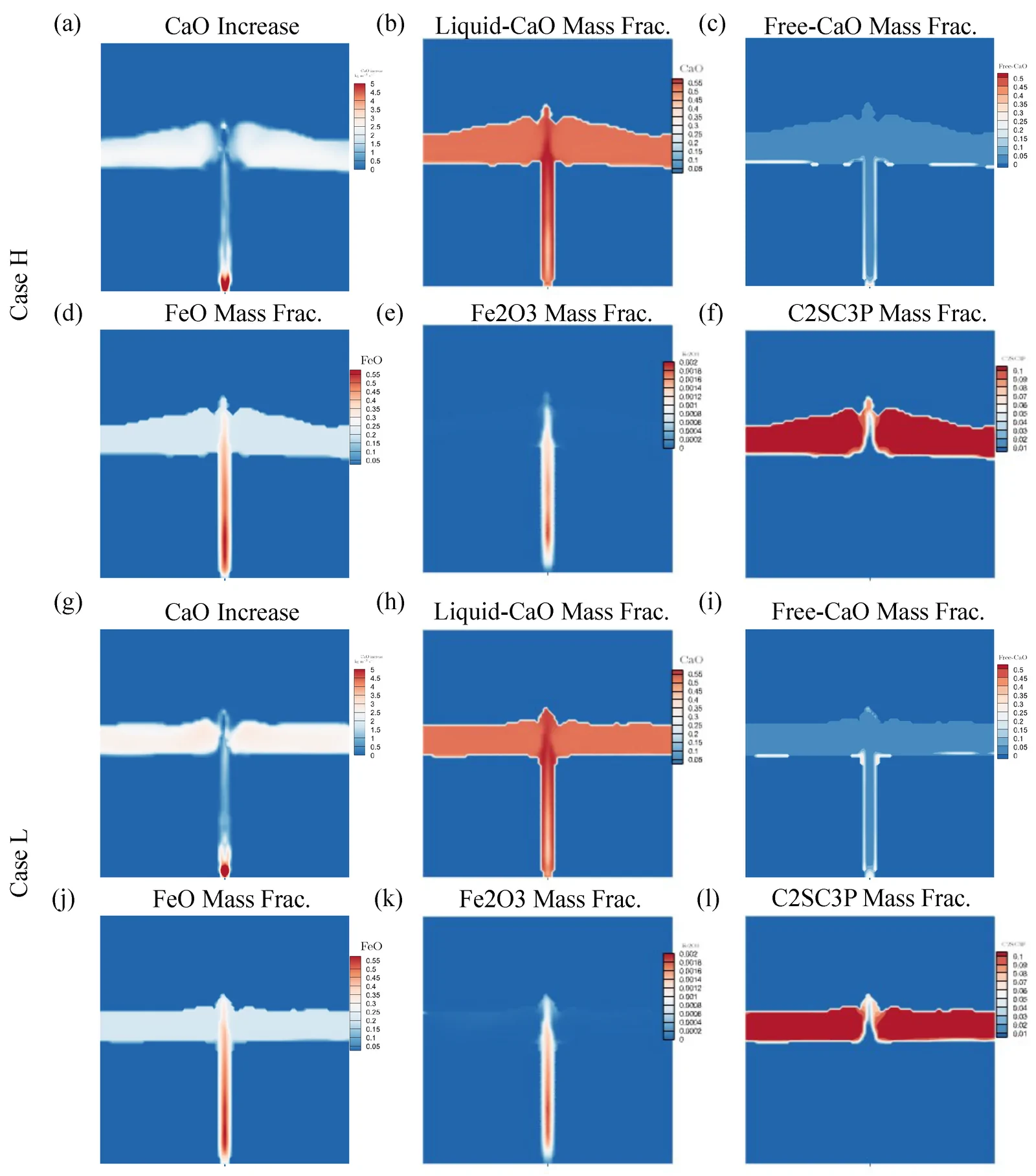
CaO, iron-oxide and $C_{2}S$–$C_{3}P$ fields at t=4.0s. Panels (**a**–**f**) show Case H and panels (**g**–**l**) show Case L. Within each case, the panels show, in order, the CaO-increase source, liquid-CaO mass fraction, free-CaO mass fraction, FeO mass fraction, ${\mathrm{Fe}}_{2}O_{3}$ mass fraction, and $C_{2}S$–$C_{3}P$ mass fraction. The CaO-increase source is in kg $m^{-3}s^{-1}$; all composition fields are dimensionless. Corresponding Case H and Case L panels use identical scales.

Figure 6 separates the CaO-increase field from the liquid-CaO and free-CaO inventories. CaO enters along the bottom-blowing column and then spreads through the slag layer. Liquid CaO is continuous across much of that layer, whereas free CaO is concentrated near the lower boundary of the slag and around the plume shoulders. The distributions are similar in form in the two cases, but the disturbed region is broader in Case H. Solid-CaO reaction and dissolution studies show why delivery and utilisation must be separated: wetting by FeO-rich slag and the resistance of the reaction layer control whether added CaO enters the liquid inventory [20,44,55].

The same figure shows that FeO is distributed through the slag layer and the injection column, while ${\mathrm{Fe}}_{2}O_{3}$ is concentrated mainly along the oxygen-bearing plume. The modelled $C_{2}S$–$C_{3}P$ inventory occupies the slag layer but is interrupted and redistributed near the plume. This field represents the available fixation phase. It is not a direct measure of phosphorus removal. Local fixation also requires newly generated $P_{2}O_{5}$ and a slag–metal transfer pathway.

**Figure 7:**
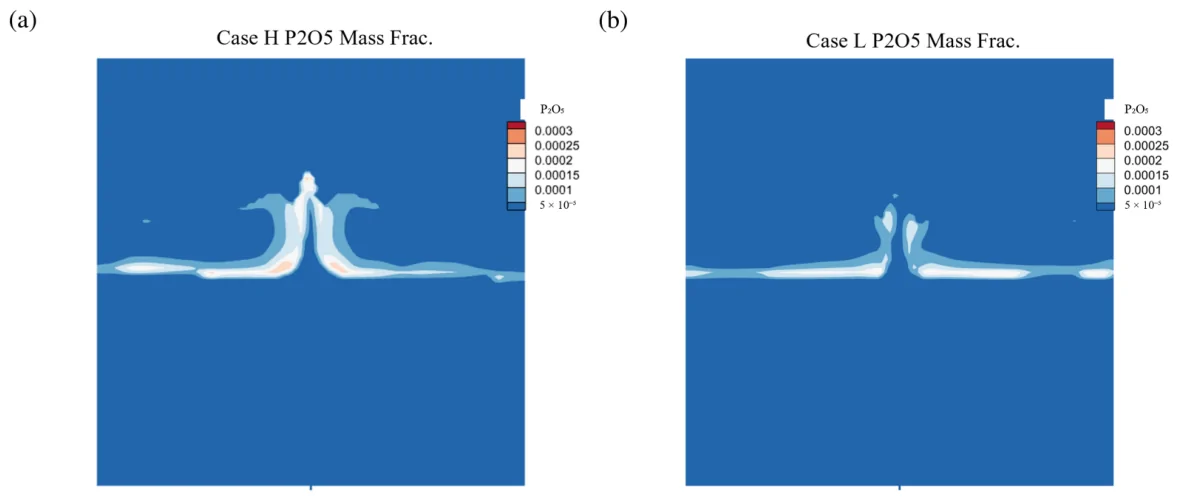
Calculated $P_{2}O_{5}$ mass fraction at t=4.0s for (**a**) Case H and (**b**) Case L. The mass fraction is dimensionless, and both panels use the same colour scale.

$P_{2}O_{5}$ is shown separately in Figure 7 so that its narrow enrichment zone is not lost in a dense composite figure. In both cases, the largest calculated $P_{2}O_{5}$ mass fractions lie along the upper slag–metal interface and the shoulders of the lifted plume. Case H has a broader vertical connection to the plume, while Case L shows a narrower interface-aligned band. $P_{2}O_{5}$ is not enriched continuously along the full bottom gas column. The result is consistent with a model in which CaO delivery, ${\mathrm{FeO}}_{x}$ oxygen supply, and phosphorus oxidation must meet near an effective slag–metal contact before fixation can occur. Solid-solution and interfacial-compound studies support the role of $C_{2}S$–$C_{3}P$ in retaining phosphate once it has entered the slag [13,15,63].

**Figure 8:**
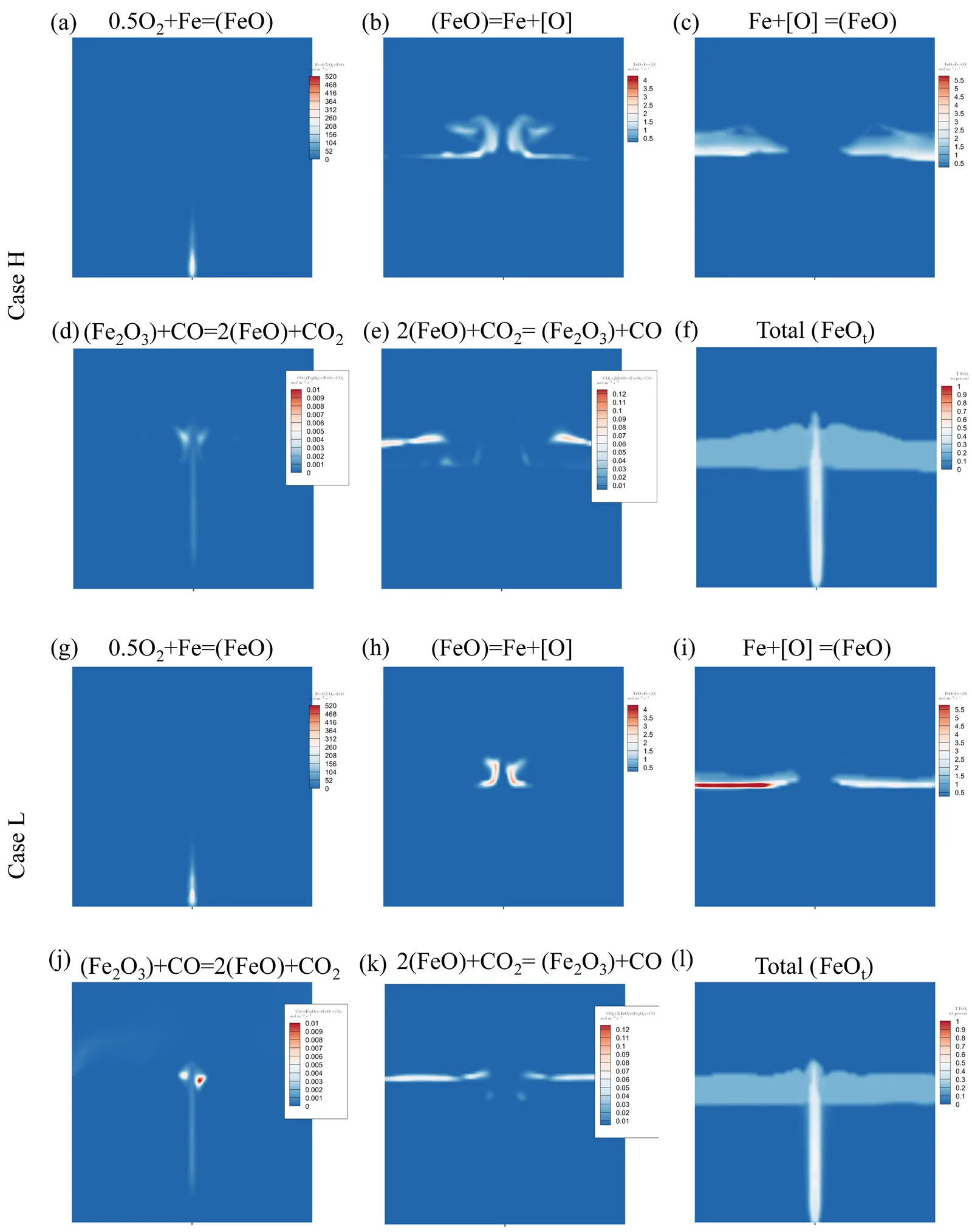
Individual ${\mathrm{FeO}}_{x}$ reaction channels and total ${\mathrm{FeO}}_{t}$ at t=4.0s. Panels (**a**–**f**) show Case H and panels (**g**–**l**) show Case L. Within each case, the panels show, in order, gas–metal FeO formation, FeO oxygen release, metal–slag FeO formation, ${\mathrm{Fe}}_{2}O_{3}$ reduction by CO, FeO oxidation by ${\mathrm{CO}}_{2}$, and total ${\mathrm{FeO}}_{t}$. Individual reaction rates are in mol $m^{-3}s^{-1}$, and total ${\mathrm{FeO}}_{t}$ is a dimensionless mass fraction. Corresponding Case H and Case L panels use identical scales.

**Figure 9:**
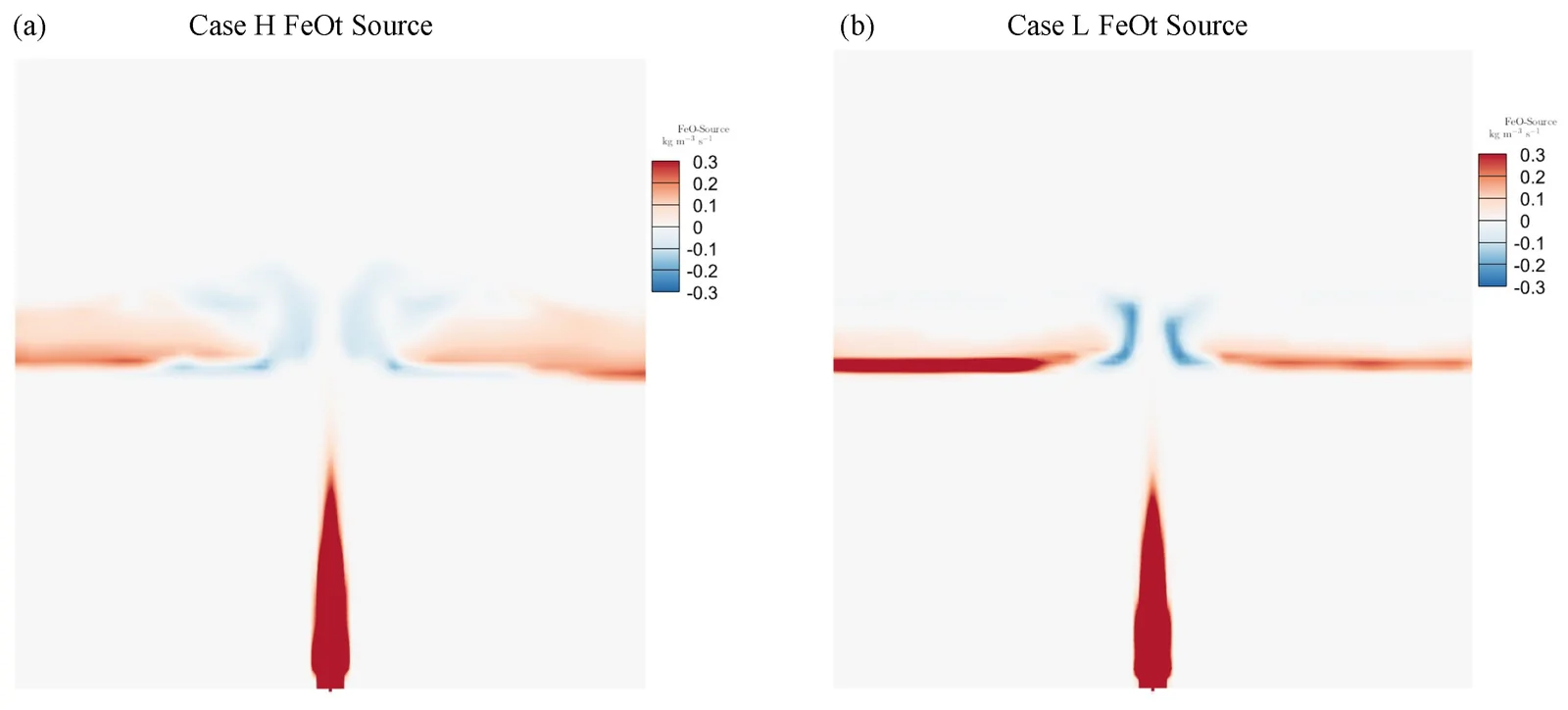
Signed net ${\mathrm{FeO}}_{t}$ mass source at t=4.0s for (**a**) Case H and (**b**) Case L, in kg $m^{-3}s^{-1}$. Both panels use the same diverging scale.

Figure 8 and Figure 9 separate the individual ${\mathrm{FeO}}_{x}$ channels from their net effect. Near the inlet, $0.5O_{2}+Fe=\left(FeO\right)$ supplies FeO. FeO can then release dissolved oxygen to the metal or be regenerated by Fe+[O]. In the upper mixed layer, the Fe(II)/Fe(III) balance is coupled to CO and ${\mathrm{CO}}_{2}$, so ${\mathrm{Fe}}_{2}O_{3}$ reduction and FeO reoxidation occur in neighbouring regions. The total ${\mathrm{FeO}}_{t}$ inventory therefore records the history of several reactions rather than one formation step. Phosphate-capacity and basic oxygen furnace (BOF) partition studies identify ${\mathrm{FeO}}_{t}$, basicity, and $P_{2}O_{5}$ activity as coupled thermodynamic controls [2,64,65].

The signed net ${\mathrm{FeO}}_{t}$ source in Figure 9 is positive along the injection column but changes sign around the upper interface. In Case H, a broad negative region appears around the central interface shoulders, consistent with reduction or transfer out of the local ${\mathrm{FeO}}_{t}$ inventory in the CO-rich plume. Case L retains a stronger positive band along the slag–metal layer, with the negative source confined more closely to the plume shoulders. The relevant distinction is therefore not whether FeO is formed near the inlet, but where the net ${\mathrm{FeO}}_{t}$ source remains positive after gas–metal supply, metal–slag exchange, and gas–slag redox are assembled. Composition adjustment can improve equilibrium dephosphorisation capacity [66,67], but the present fields show that this capacity contributes only where ${\mathrm{FeO}}_{t}$ persists in the reaction zone.

### 3.4. Selectivity Among Decarburisation, Desiliconisation, and Dephosphorisation

**Figure 10:**
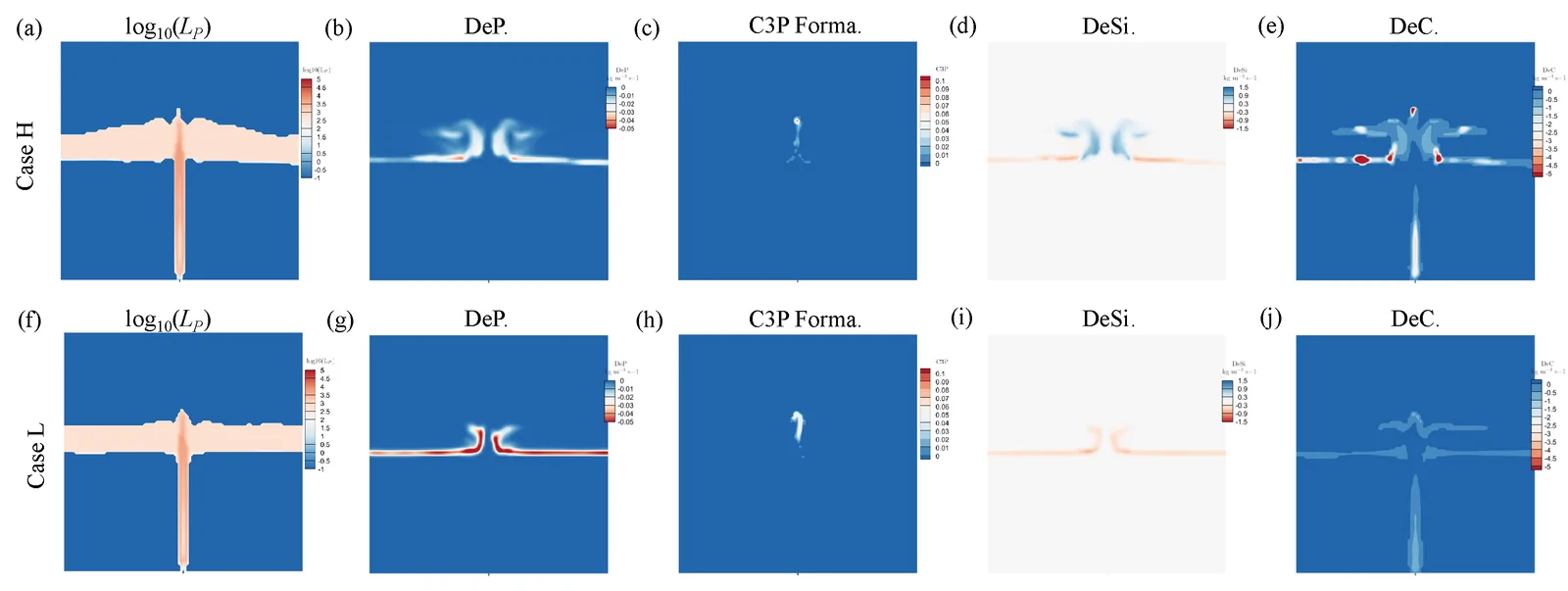
Reaction-selectivity fields at t=4.0s. Panels (**a**–**e**) show Case H and panels (**f**–**j**) show Case L. Within each case, the panels show, in order, $\log_{10}L_{P}$, dephosphorisation, $C_{2}S$–$C_{3}P$ formation, desiliconisation/resiliconisation, and decarburisation; $\log_{10}L_{P}$ is dimensionless; the reaction fields are signed mass sources in kg $m^{-3}s^{-1}$. Corresponding Case H and Case L panels use identical scales.

Figure 10 compares local phosphorus-partition capacity with the calculated reaction sources. The field $\log_{10}L_{P}$ is an equilibrium tendency based on slag composition, temperature, and oxidation state. It is not the dephosphorisation rate. High $L_{P}$ extends through parts of the slag and the injection column in both cases, while the dephosphorisation source is confined mainly to the slag–metal interface. Classical and BOF phosphorus-distribution models describe the partitioning trend [2,6], but the present source map also requires transport and local inventory.

The $C_{2}S$–$C_{3}P$ formation source is even more localised, appearing near the upper plume where $P_{2}O_{5}$ formation and fixation capacity overlap. The desiliconisation field changes sign around the interface, which indicates neighbouring oxidation and return pathways under the adopted source convention. The decarburisation field occupies a much larger fraction of the plume in Case H. Case L has a weaker carbon sink and a stronger interface-aligned dephosphorisation band. Oxygen entering carbon-rich metal is preferentially consumed by [C] to form CO [51]; however, the difference between these cases also contains the effects of Si and initial dissolved O. Metal-droplet studies likewise show that CO generation can renew interfacial area while changing metal-side transport [47].

**Figure 11:**
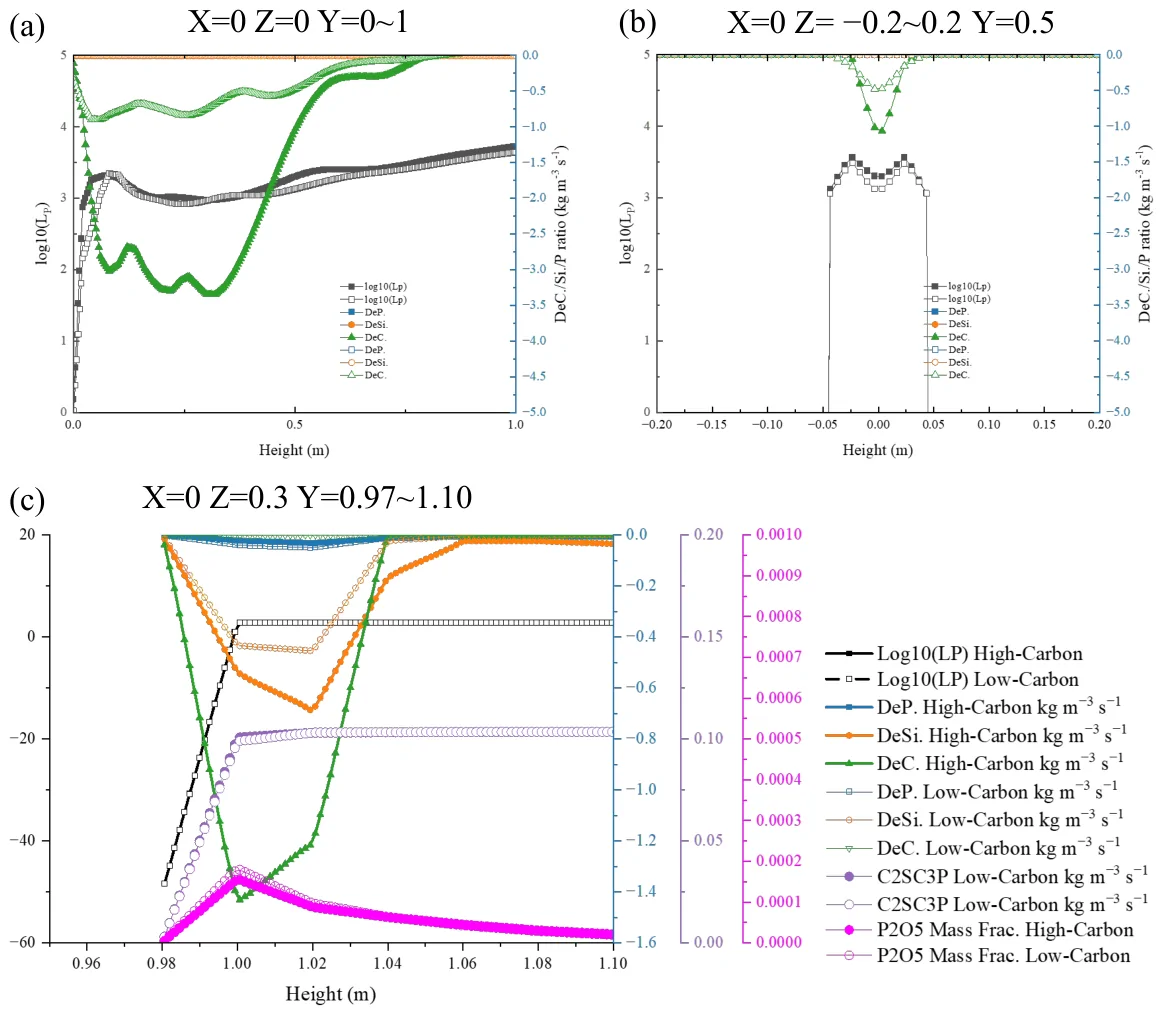
Path profiles of reaction capacity and source terms at t=4.0s. Panel (**a**) follows the central vertical axis (x=z=0, 0≤y≤1m); panel (**b**) crosses the plume at y=0.5m and −0.2≤z≤0.2m; panel (**c**) samples the upper slag–metal region at z=0.3m and 0.97≤y≤1.10m; $\log_{10}L_{P}$ is dimensionless, reaction sources are in kg $m^{-3}s^{-1}$, and $P_{2}O_{5}$ is a dimensionless mass fraction. The embedded High-Carbon and Low-Carbon legend entries denote the complete Case H and Case L composition states, not a carbon-only comparison.

The path profiles in Figure 11 test whether the spatial conclusions survive one-dimensional sampling. Along the central axis, $\log_{10}L_{P}$ remains high over much of the bath, whereas the dephosphorisation source is close to zero outside the upper interface. Decarburisation is the dominant sampled sink along the Case H axis. Across the mid-height transverse line, the reaction sources are confined to the narrow central plume. The upper-interface profile shows the strongest overlap among the negative P source, $C_{2}S$–$C_{3}P$ formation, and the $P_{2}O_{5}$ maximum. This overlap is concentrated near the disturbed interface rather than distributed through the bath.

The profiles also expose the model’s central limitation. A high calculated $L_{P}$ does not create P removal unless oxygen supply, slag–metal contact, and P transfer and fixation are present at the same location. Kinetic work on CaO–FeO–SiO_2_ slags shows that metal-side and slag-side transport are coupled [42,45]. Lime must also enter a usable slag environment before it contributes to fixation [55]. These requirements explain why the calculated P sink follows the interface and $P_{2}O_{5}$ band rather than the full region of high $L_{P}$.

Case H and Case L thus differ in the balance between interface renewal and oxygen-potential retention. Case H has stronger stirring and decarburisation, but more of the shared oxygen inventory enters CO formation and ${\mathrm{FeO}}_{x}$ buffering. Case L has weaker plume renewal but retains more ${\mathrm{FeO}}_{x}$ and $P_{2}O_{5}$ near the interface. Since the initial Si and dissolved O are not controlled, this is a comparison of two operating states rather than a carbon-only test. Industrial staged, double-slag, and retained-slag practices also manage the separation between phosphorus removal, decarburisation demand, and available slag capacity [68,69,70].

### 3.5. Macroscopic Rates, Flow Sustainment, and Thermal Feedback

For element *i*, the source term is integrated over the full domain and converted into the change rate of metal-phase mass fraction:

(13) $$R_{i}^{\mathrm{macro}}=-6000\;\frac{\int_{\Omega }S_{i}\;dV}{\int_{\Omega }\rho_{m}\alpha_{m}\;dV}.$$

In Equation (13), $S_{i}$ is the local mass source of element *i*, Ω is the computational domain, and $\rho_{m}\alpha_{m}$ gives the local metal mass per unit mixture volume. The numerator becomes ${\mathrm{kg s}}^{-1}$ after volume integration, and the denominator is the total metal-phase mass. The factor 6000=100×60 converts mass-fraction change per second to ${\mathrm{wt\% min}}^{-1}$.

**Figure 12:**
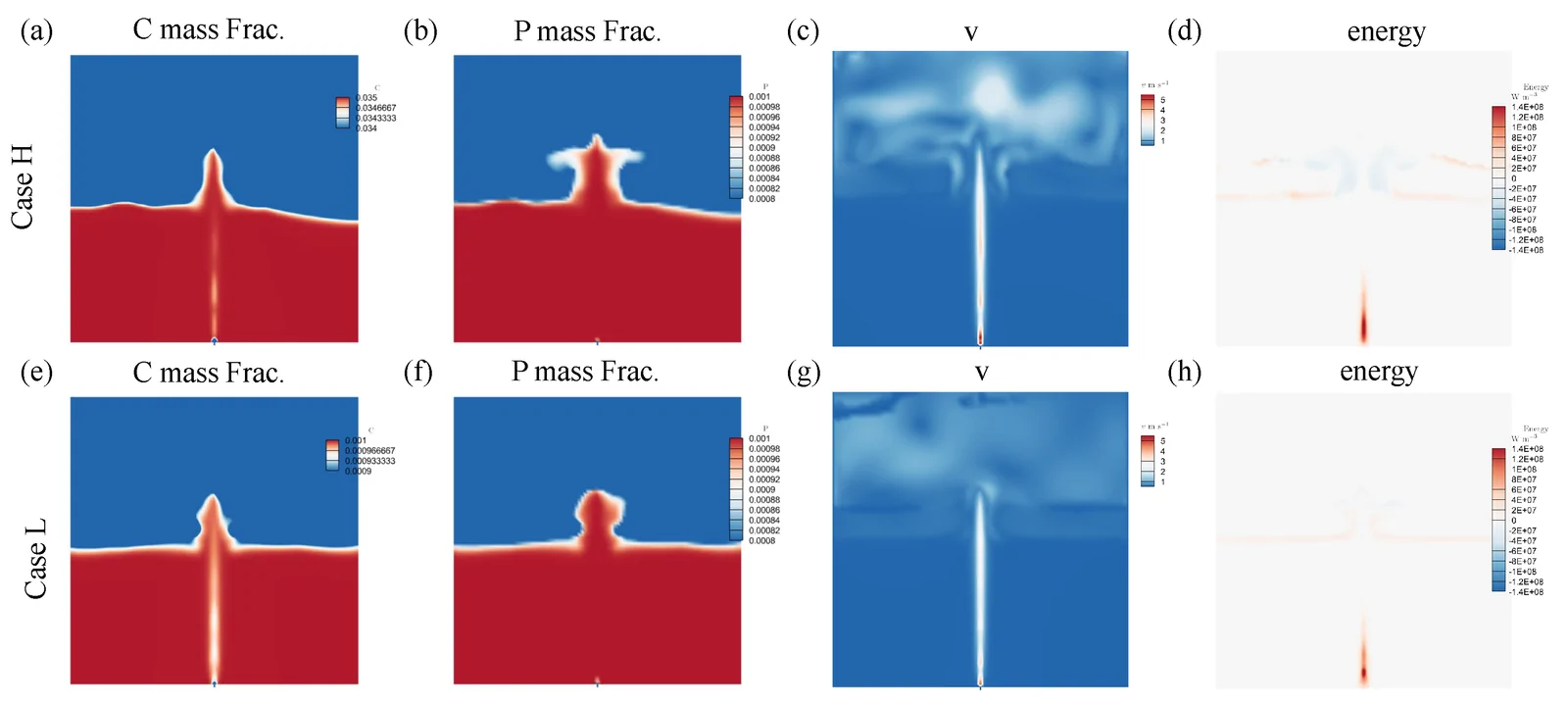
Composition, velocity, and energy-source fields at t=4.0s. Panels (**a**–**d**) show Case H and panels (**e**–**h**) show Case L. Within each case, the panels show C mass fraction, P mass fraction, velocity magnitude, and volumetric energy source. C and P are dimensionless metal-phase mass fractions, velocity is in m $s^{-1}$, and energy source is in W $m^{-3}$. Corresponding P, velocity, and energy panels use identical scales; the C ranges differ because of the different initial C mass fractions.

Figure 12 retains the spatial fields used to interpret the integral. Carbon loss in Case H follows the injection axis and extends into the lifted upper plume. The corresponding Case L carbon field changes over a much smaller initial range. In both cases, the P field changes most clearly near the slag–metal interface rather than throughout the metal bath. The velocity field follows the gas column and then spreads into the upper circulation cells. The energy source is strongest near the inlet and remains detectable around the upper mixed layer. Paired P, velocity, and energy panels use the same scales; the C scales differ because the initial C mass fractions differ by a factor of 35.

**Figure 13:**
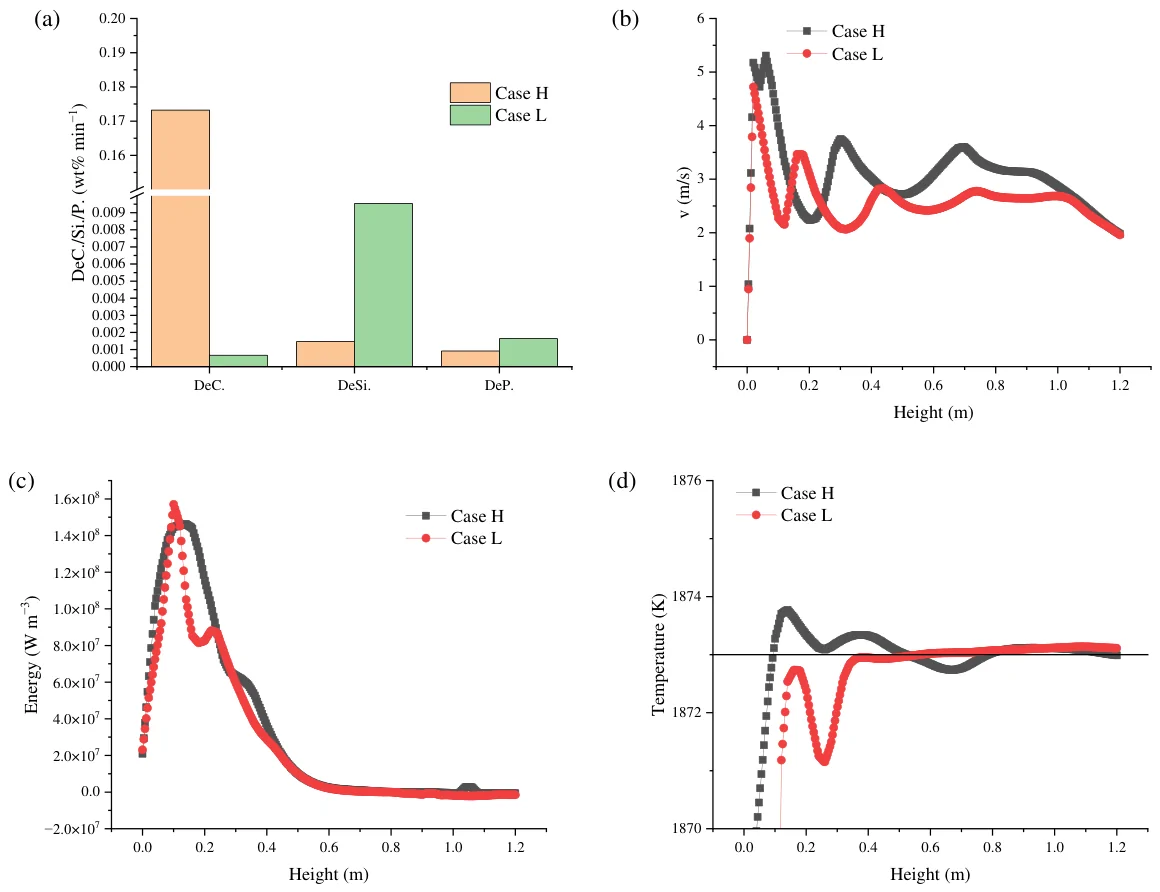
Integrated rates and axial profiles at t=4.0s. Panel (**a**) gives domain-integrated decarburisation, desiliconisation, and dephosphorisation rates in wt% $\min^{-1}$ for Cases H and L. Panels (**b**–**d**) compare axial velocity in m $s^{-1}$, volumetric energy source in W $m^{-3}$, and temperature in K as functions of height in m.

The integrated rates in Figure 13a follow the local source maps. Decarburisation dominates Case H, while the Case L decarburisation rate is much smaller. Desiliconisation and dephosphorisation are more visible in Case L, although the integrated P rate remains small compared with Case H decarburisation. The P value is a full-domain integral and cannot be assigned to one particle trajectory or one section of the gas column. Industrial $O_{2}$–CaO bottom-blowing, double-slag operation, and BOF reviews likewise identify oxygen supply, slag inventory, ${\mathrm{FeO}}_{t}$, and temperature as coupled controls [1,37,71].

The axial profiles in Figure 13b–d show how reaction feeds back into flow and heat. The velocity rises sharply above the inlet and remains in the order of several ${\mathrm{m s}}^{-1}$ through much of the plume. Case H generally maintains the higher velocity because its larger CO source adds gas volume and buoyancy. The energy source peaks in the lower plume, decays through the middle bath, and shows only small upper-layer features. Temperature changes remain within a few kelvin of the initial bath value over this short transient. Bottom-blowing studies relate plume buoyancy to circulation and mixing [56,57]; gas–slag–metal emulsion calculations likewise place the reaction zone on the moving plume rather than on a fixed surface [72].

Reported oxygen-steelmaking hot-spot temperatures are much higher than the response in Figure 13d, but they describe a different physical zone. Values of 2000–2500 °C usually refer to a top-blown oxygen-jet cavity where oxygen impinges directly on the metal surface and reaction heat is concentrated [73,74]. Zone heat-balance and droplet models can predict 1900–2090 °C, with higher local peaks, because they prescribe a hot-spot zone and calibrated heat-transfer paths [75]. Those values are useful for top-blown BOF analysis but are not a direct expectation for every cell near a bottom oxygen inlet.

The present calculation solves a moving, reaction-limited gas–slag–metal field rather than imposing a fixed hot-spot temperature. The plotted heat source is an instantaneous volumetric rate, not the cumulative heat retained in a stationary control volume. A local temperature increment scales with the retained residence time, $\Delta T∼\eta q^{\prime \prime \prime }\tau_{\mathrm{res}}/\left(\rho c_{p}\right)$, where $q^{\prime \prime \prime }$ is the volumetric heat-release rate, $\tau_{\mathrm{res}}$ is the time over which the same material volume remains in the reactive region, and η<1 represents heat carried away by convection, turbulent dispersion, and interphase exchange. In the bottom plume, $\tau_{\mathrm{res}}$ is short: liquid steel is swept upward, CO generation accelerates the plume, gas–metal slip renews the interface, and the 300 K injected gas introduces an initial cooling sink. Therefore, even when $q^{\prime \prime \prime }$ locally reaches the order of $10^{8}$${\mathrm{W m}}^{-3}$, much of the released heat is transported with the flowing steel and dispersed into a larger bath volume before it can accumulate as a persistent superheated cell.

The thermal field is also limited chemically. Heat release is constrained by local $O_{2}$, carbon competition, ${\mathrm{FeO}}_{x}$ conversion, slag–metal contact, and CaO-assisted reaction capacity rather than by a fixed stoichiometric oxygen-consumption rate at the tuyere. Strong decarburisation generates CO and buoyancy but does not necessarily preserve the oxygen potential and ${\mathrm{FeO}}_{x}$ needed for P fixation. Within the selected thermal closure and the 4.0 s window, the small temperature change in Figure 13d is consistent with a convective, reaction-limited bottom plume. Thermal feedback mainly changes gas generation, slip, and interface renewal near the plume. The calculated P sink follows the spatial overlap of oxygen potential, slag–metal contact, and phosphate-fixation capacity.

### 3.6. Mechanistic Synthesis

The split fields give a consistent sequence within the selected closures. Oxygen and CaO enter through the bottom boundary. The lower plume forms FeO and converts dissolved carbon to CO, while particles release CaO only along their tracked paths. Gas generation expands the plume and renews gas–metal contact. Near the upper layer, gas–slag redox redistributes ${\mathrm{FeO}}_{x}$, and the deformed phase field creates a finite slag–metal contact zone. P removal becomes appreciable only where that contact coincides with retained ${\mathrm{FeO}}_{x}$, $P_{2}O_{5}$ production, and $C_{2}S$–$C_{3}P$ fixation.

**Figure 14:**
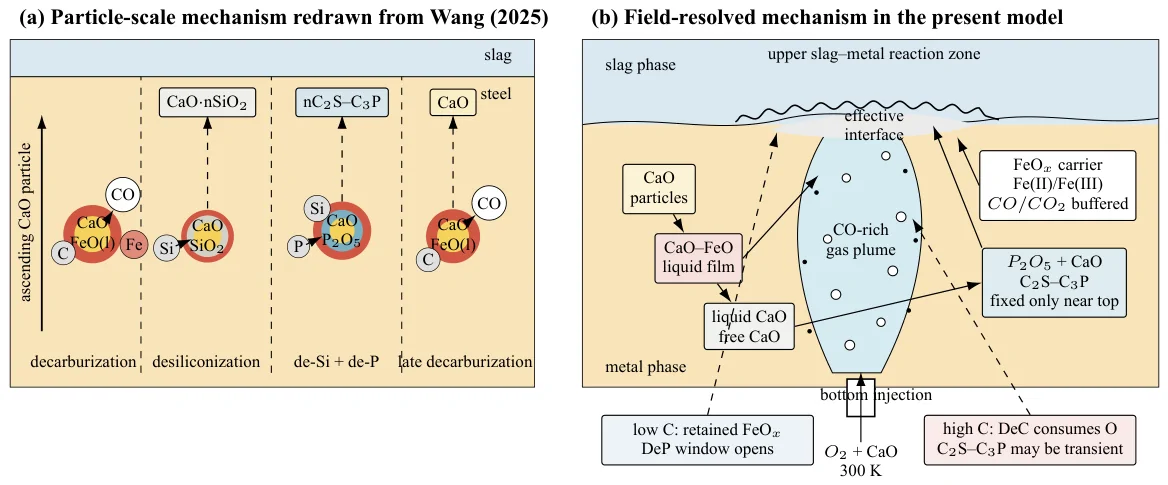
Schematic interpretation of gas–slag–metal coupled dephosphorisation during bottom-blown oxygen–CaO powder injection. Panel (**a**) is redrawn from Wang Chunyang’s particle-flotation and reaction schematic for a bottom-blown $O_{2}$–$CO_{2}$–lime-powder process [76]. Panel (**b**) summarises the field-resolved sequence inferred from the present model: CaO–FeO liquid-film formation and CaO release occur along the particle path, whereas sustained P removal requires overlap among retained ${\mathrm{FeO}}_{x}$, the effective slag–metal interface, $P_{2}O_{5}$ production, and $C_{2}S$–$C_{3}P$ fixation. The “high C” and “low C” annotations describe the two complete Case H and Case L composition states; because Si and initial dissolved O also differ, the schematic does not isolate a carbon-only effect.

Figure 14 places the particle-scale interpretation from the literature beside the field-resolved mechanism inferred under the present closures. Particle-scale descriptions of bottom-blown oxygen–lime refining propose CaO–FeO liquid-film formation, CaO–SiO_2_ reaction layers, CaO–P_2_$O_{5}$ reaction, and $C_{2}S$–$C_{3}P$ fixation during flotation [76]. Bottom-blown lime–oxygen and powder-injection experiments support rapid reaction after contact with FeO-rich liquid slag, but they also show that residence path and coupling with the slag–metal interface affect utilisation [35,77,78]. A local phosphorus-bearing calcium-silicate indicator therefore records a local step. It does not by itself demonstrate a sustained bath-scale removal channel.

Within the selected closure set, macroscopic dephosphorisation requires [P] transfer, ${\mathrm{FeO}}_{x}$ oxygen supply, CaO-particle dissolution, $P_{2}O_{5}$ generation, and $C_{2}S$–$C_{3}P$ fixation to overlap in an effective slag–metal contact zone. This overlap is less persistent in Case H: more of the supplied oxygen enters the calculated decarburisation channel, and the resulting CO is associated with lower ${\mathrm{FeO}}_{x}$ retention along the flotation path. A local P-oxidation or $C_{2}S$–$C_{3}P$ source can still occur, but the model also permits redissolution and rephosphorisation in a later reducing region. A phosphorus-enriched phase near a particle is therefore evidence of a local reaction in the model, not proof of sustained bath-scale dephosphorisation. Whether this behaviour applies to a high-carbon process stage must be tested with controlled-composition simulations and matched experiments.

This distinction is also relevant when interpreting experiments. Endpoint P in an oxygen–lime-powder trial can reflect top-slag reaction, furnace oxygen potential, slag volume and basicity, mixing, and temperature history, as well as reactions along the powder path. The two calculations do not separate those contributions. They show only that, under the present assumptions, the calculated P sink becomes appreciable where the CaO–FeO reaction environment, ${\mathrm{FeO}}_{x}$ retention, and the slag–metal interface overlap. This is a hypothesis for targeted validation rather than evidence for a general operating strategy. In particular, the present results do not quantify the direct powder-flotation contribution in hot metal or establish that one converter stage is preferable to another.

## 4. Conclusions

This study formulates an Euler–Euler–DPM framework for gas–slag–metal–particle reactions during bottom-blown oxygen–CaO-powder injection. The framework places gas oxygen potential, ${\mathrm{FeO}}_{x}$ conversion, particle dissolution, slag–metal transfer, $P_{2}O_{5}$ generation, $C_{2}S$–$C_{3}P$ fixation, and reaction heat in one conservative source-term system. Its purpose in the present work is to examine where these modelled processes overlap in a non-uniform bath.

For the selected closure set, the active P-removal source is concentrated in the upper slag–metal mixing region rather than throughout the bottom gas column. Case H develops a broader CO-rich plume and larger gas–metal area, while Case L retains more ${\mathrm{FeO}}_{x}$ and dissolved oxygen near the slag–metal interface. Since the two states also differ in Si and initial dissolved O, this contrast cannot be attributed to carbon alone. A controlled composition pair is needed to quantify a carbon effect.

The calculations also distinguish CaO delivery from phosphorus fixation. Particle mass enters the slag inventory only after contact with an effective slag or FeO-rich region. The resulting CaO contributes to the calculated P sink only where ${\mathrm{FeO}}_{x}$, $P_{2}O_{5}$ production, and the $C_{2}S$–$C_{3}P$ closure coexist. A high local $L_{P}$ or a phosphorus-bearing solid indicator therefore represents capacity within the model, not by itself a verified macroscopic removal rate.

These findings are conditional. They are based on two confounded initial composition states, one production mesh supplemented by one coarse-grid comparison, one reported production time step, one set of interfacial and kinetic closures, and a short transient ending at the nominal time of 4.0 s. The present figures should be read as spatial hypotheses generated by the model, not as validated process predictions or a basis for recommending an industrial operating window.

Quantitative validation against matched plume, gas-holdup, CaO-dissolution, decarburisation, or phosphorus-removal data remains necessary. A formal three-grid convergence/GCI assessment, time-step independence, closure sensitivity, time histories, and controlled-composition calculations are also required for quantitative prediction. Until those studies are completed, the framework is best used to organise coupled transport and reaction mechanisms and to identify measurements that can test the predicted reaction-zone structure.

## Figures and Tables

**Table 1:** Reaction rates, closure reactions, and the corresponding changes in the gas, slag, and metal compositions. Upward and downward arrows denote an increase and a decrease, respectively, in the listed phase inventory; ↔ denotes reversible exchange.

Rate	Closure Reaction	Gas-Phase Changes	Metal-Phase Changes	Slag-Phase Changes	Effect
$r_{FeO}^{form}$	$Fe+\frac{1}{2}O_{2}\to FeO$	$O_{2}$↓	Fe is consumed as a balance metal	FeO↑	FeO is generated at the gas–metal contact, and the product is included in the slag-oxide inventory.
$r_{FeO}^{ms}$	Fe+[O]→FeO	no direct changes	[O]↓,Fe↓	FeO↑	Metal dissolved oxygen continues to generate FeO at the metal–slag interface.
$r_{O_{2}}^{diss}$	$O_{2}\to 2\left[O\right]$	$O_{2}↓$	[O]↑	no direct changes	The inlet oxygen directly enters the metal dissolved oxygen reservoir.
$r_{FeO}^{diss}$	FeO→Fe+[O]	no direct changes	[O]↑	FeO↓	FeO in the slag supplies oxygen to the metal when it is deficient in oxygen.
$r_{CO}^{ox}$	$CO+\frac{1}{2}O_{2}\to CO_{2}$	$CO↓,O_{2}↓,CO_{2}↑$	no direct changes	no direct changes	The CO produced by decarburisation continues to consume oxygen and increase the ${\mathrm{CO}}_{2}$ partial pressure.
$r_{CO_{2}}^{diss}$	$CO_{2}↔CO+\left[O\right]$	$CO_{2}↔CO$	[O]↔	no direct changes	${\mathrm{CO}}_{2}$ cracks for oxygen and CO–[O] recombination closes at the same signed rate.
$r_{C}^{ox}$	[C]+[O]→CO	CO↑	[C]↓,[O]↓	no direct changes	The decarburisation reaction connects metallic carbon to gas-phase CO.
$r_{Si}^{ox}$	$\left[Si\right]+2\left[O\right]\to SiO_{2}$	no direct changes	[Si]↓,[O]↓	$SiO_{2}↑$	Silicon oxidation preferentially consumes dissolved oxygen and increases acidic oxides.
$r_{P}^{fwd}$	$\left[P\right]+2.5\left[O\right]\to 0.5P_{2}O_{5}$	no direct changes	[P]↓,[O]↓	$P_{2}O_{5}↑$	One forward flux controlled in series by the $L_{P}$ driving force, the effective metal–slag kinetic coefficient, the local P inventory, and the shared oxygen allocation.
$r_{P}^{rev}$	$0.5P_{2}O_{5}\to \left[P\right]+2.5\left[O\right]$	no direct changes	[P]↑,[O]↑	$P_{2}O_{5}↓$	Limited rephosphorisation from the mobile phosphate pool when the local $L_{P}$ relation favours return to the metal.
$r_{Fe_{2}O_{3}}^{O_{2}}$	$2FeO+\frac{1}{2}O_{2}\to Fe_{2}O_{3}$	$O_{2}↓$	no direct changes	$FeO↓,Fe_{2}O_{3}↑$	Gas–slag oxygen transfer raises the ferric-iron fraction in the slag.
$r_{CO_{2}-FeO}$	$2FeO+CO_{2}\to Fe_{2}O_{3}+CO$	$CO_{2}↓,CO↑$	no direct changes	$FeO↓,Fe_{2}O_{3}↑$	${\mathrm{CO}}_{2}$ oxidises FeO to higher-valence iron while generating CO.
$r_{CO-Fe_{2}O_{3}}$	$CO+Fe_{2}O_{3}\to CO_{2}+2FeO$	$CO↓,CO_{2}↑$	no direct changes	$Fe_{2}O_{3}↓,FeO↑$	CO from decarburisation reduces ferric iron and closes the indirect post-combustion loop.
$r_{CaO}^{free}$	$CaO_{free}↔CaO_{liq}$	no direct changes	no direct changes	$CaO_{free}↔CaO$	The internal phase distribution of CaO particles after dissolution does not produce a new fluid phase.
$r_{C2S}$	$2CaO+SiO_{2}\to C_{2}S$	no direct changes	no direct changes	$CaO↓,SiO_{2}↓,C_{2}S--C_{3}P↑$	A dicalcium silicate skeleton is generated.
$r_{C3P}$	$3CaO+P_{2}O_{5}\to C_{3}P$	no direct changes	no direct changes	$CaO↓,P_{2}O_{5}↓,C_{2}S--C_{3}P↑$	Fixes $P_{2}O_{5}$ and increases the $C_{3}P$ end-member share in the solid phase.
${\dot{m}}_{CaO}^{p}$	$CaO_{p}\to CaO_{liq}/CaO_{free}$	no direct changes	no direct changes	$CaO↑,CaO_{free}↑$	The first-order particle mass-loss source in the effective slag phase or FeO reaction film is split into two parts: CaO entering the liquid slag directly and CaO stored temporarily as free CaO.

**Figure A1:**
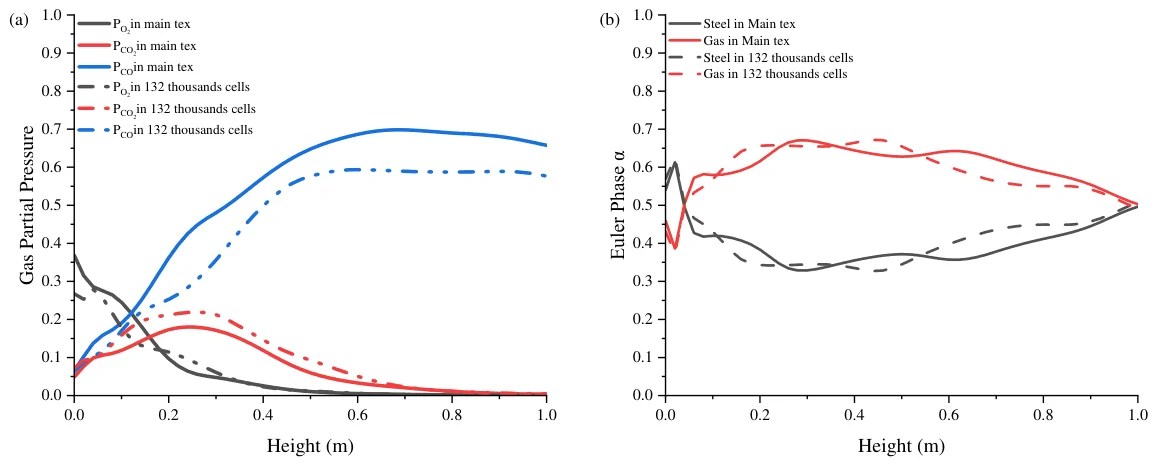
Case H qualitative two-grid comparison along the central vertical line (x=z=0) at t=4.0s. Solid lines denote the 1,020,102-cell production mesh, and dashed lines denote the approximately 132,000-cell mesh. (**a**) Axial $O_{2}$, $CO_{2}$, and CO partial pressures, reported in atm using the same definition as Figure 5. (**b**) Selected axial steel- and gas-phase Eulerian volume fractions, α, which are dimensionless. Height is measured from the bottom inlet. The slag volume fraction is not included in the author-supplied comparison graphic.

## Data Availability

The original contributions presented in this study are included in the article. Further inquiries can be directed to the corresponding authors. The Euler–Euler–DPM code and calculation files, together with a supplementary VOF calculation scheme, are mirrored at https://github.com/Zhenkeaidai/CFD-DeP-C-Si-In-Steelmaking.git (accessed on 12 July 2026). The VOF files document an alternative calculation route and were not used to generate the Euler–Euler results reported in this manuscript.

## Appendix A. Thermodynamic and Reaction Closures

### Appendix A.1. Thermodynamic Equilibrium Functions and Constraints

The thermodynamic closure does not embed a full multivariate database in the flow calculation. Instead, it reduces the relationships needed by the local source terms to a set of target quantities: gas partial pressure and oxygen potential, iron-oxide valence state, equivalent slag alkalinity and $FeO_{t}$, phosphorus distribution ratio, liquid CaO capacity, $C_{2}S$–$C_{3}P$ fixation capacity, and dilute-solution metal activities. Phosphorus distribution and slag–metal balance are constrained by CaO–SiO_2_–FeO–P_2_$O_{5}$ and BOF slag experiments and empirical relationships [6,7,8,9,65,79,80,81,82,83,84,85]. Iron-oxide activity and oxygen potential are constrained by FeO, ${\mathrm{Fe}}_{2}O_{3}$, and multicomponent slag-activity studies [52,64,66,86,87,88]. These relations define local equilibrium targets and reaction directions; the converted amount remains controlled by kinetics and inventory limits.

These algebraic relations are evaluated inside a user-defined function (UDF) during the transient computational fluid dynamics (CFD) calculation. At each flow-field update, the function reads the local transported phase composition, pressure, and energy-equation temperature, evaluates the equilibrium targets and finite kinetic rates, applies the shared inventory limits, and returns species, phase-mass, and energy source terms to the next solver update. ANSYS Fluent advances the transport equations, but no external thermodynamic package and no separate equilibrium solver are called. The closure therefore supplies local reaction targets rather than a simultaneous full-database equilibrium calculation.

The same reduction is used for CaO and phosphorus-rich solids. CaO’s entry into liquid slag is controlled mainly by FeO fluxing, ${\mathrm{SiO}}_{2}$ crust formation, $P_{2}O_{5}$ fixation, and temperature. The $2CaO\cdot SiO_{2}$ and $3CaO\cdot P_{2}O_{5}$ solid-solution limits determine whether $P_{2}O_{5}$ is locked into a low-activity state [10,11,12,13,14,15,20,29,63,89,90,91,92,93,94]. The virtual solid phase is therefore an accounting term, not an additional fluid phase. It deducts CaO, ${\mathrm{SiO}}_{2}$, and $P_{2}O_{5}$ from the liquid-slag inventory and permits only limited dissolution or phosphorus recovery when the local oxygen potential or CaO capacity decreases.

#### Appendix A.1.1. Gas Thermodynamics and Oxygen Potential

The gas-phase mass fractions are first converted into mole fractions. This relation shows that the molar amounts of $O_{2}$, CO, $CO_{2}$, and Ar jointly determine the gas composition. Molar masses use standard atomic weights from the International Union of Pure and Applied Chemistry (IUPAC) and molecular data from the NIST [49,95].

(A1) $$x_{i}=\frac{Y_{i}/M_{i}}{\sum_{j}Y_{j}/M_{j}}$$

In Equation (A1), $x_{i}$ is the mole fraction of gas-phase component *i*, $Y_{i}$ is its mass fraction, and $M_{i}$ is its molar mass.

The partial pressure follows the ideal-gas mixture relation. This relation converts the gas-phase composition into a reaction driving force, while $p_{\min}$ avoids singular values in cells with vanishing gas content or extremely low partial pressure [49,96].

(A2) $$p_{i}=\max\left(p_{\min},x_{i}p^{\circ }\right)$$

In Equation (A2), $p^{\circ }$ is the standard atmospheric pressure, and $p_{\min}$ is the lower limit of the numerical partial pressure.

The ${\mathrm{CO/CO}}_{2}$ buffer oxygen potential is derived from the CO-oxidation equilibrium [49].

(A3) $$p_{O_{2}}^{CO/CO_{2}}={\left(\frac{p_{CO_{2}}}{K_{CO,ox}p_{CO}}\right)}^{2}$$

Equation (A3) shows that even if there is no CO or ${\mathrm{CO}}_{2}$ at the inlet, the CO produced by decarburisation will be coupled with $O_{2}$ through the CO afterburning channel in Table 1, thereby changing the local oxygen potential.

The local oxygen potential used for the slag–iron-oxide equilibrium is the larger of the direct oxygen partial pressure and the $CO/CO_{2}$ buffer oxygen potential. This equation represents the effect of gas-phase composition on the oxidation state of the slag [49,52].

(A4) $$p_{O_{2}}^{slag-gas}=\max\left(p_{O_{2}},p_{O_{2}}^{CO/CO_{2}}\right)$$

In Equation (A4), $p_{O_{2}}$ comes from the local $O_{2}$ mole fraction, and $p_{O_{2}}^{CO/CO_{2}}$ comes from the $CO/CO_{2}$ equilibrium; both determine the $FeO/Fe_{2}O_{3}$ target ratio.

The CO oxidation equilibrium constant is given by the standard free energy [49].

(A5) $$K_{CO,ox}=\exp\left(-\frac{\Delta G_{CO,ox}^{\circ }}{RT}\right)$$

In Equation (A5), $\Delta G_{CO,ox}^{\circ }=-282,400+86.8T\;J\;{\mathrm{mol}}^{-1}$ is a linearised expression based on the Joint Army–Navy–Air Force (JANAF) thermochemical tables and NIST data over the stated temperature range.

The equivalent equilibrium constant for ${\mathrm{CO}}_{2}$ cleavage is also determined from the free energy [49].

(A6) $$K_{CO_{2},diss}=\exp\left(-\frac{\Delta G_{CO_{2},diss}^{\circ }}{RT}\right)$$

In Equation (A6), $\Delta G_{CO_{2},diss}^{\circ }=157,730-85.87T\;J\;{\mathrm{mol}}^{-1}$; this term allows $CO_{2}$ to supply oxygen for metal oxidation and to participate in the slag–iron-oxide balance.

All interfacial relaxation rates use the same temperature-corrected form. This equation converts the empirical rate constant at the reference temperature into the value at the model temperature and is equivalent to an Arrhenius relation normalised by $T_{\mathrm{ref}}$ [2,25].

(A7) $$k\left(T\right)=k_{\mathrm{ref}}\exp\left[-\frac{E_{a}}{R}\left(\frac{1}{T}-\frac{1}{T_{\mathrm{ref}}}\right)\right]$$

In Equation (A7), $k_{\mathrm{ref}}$ and $E_{a}$ are parameters to be calibrated. They are not interpreted as fundamental material properties, because they also reflect mixing, interface renewal, and mass transfer at the grid scale.

The thermodynamic functions and kinetic rates use the local temperature from the energy equation rather than a fixed bath temperature. The reaction calculation uses the effective value after applying the stated temperature bounds.

(A8) $$T=T_{\mathrm{eff}}$$

In Equation (A8), $T_{\mathrm{eff}}$ comes from the current control-volume temperature in the energy equation and is limited to 1200–2300K. $T_{\mathrm{bath}}=1873\;K$ is used only as the initial temperature, reference temperature, and fallback when the local temperature is invalid. At the next time step, the energy-equation solution feeds back through $T_{\mathrm{eff}}$ to the equilibrium constants, phosphorus distribution, Arrhenius rates, CaO-particle dissolution rate, and reaction enthalpy.

#### Appendix A.1.2. Slag Thermodynamics

The balance between ${\mathrm{Fe}}_{2}O_{3}$ and FeO adopts a van’t Hoff-type temperature correction. This equation allows the iron-oxide ratio to vary with temperature and oxygen potential rather than remain fixed at its initial value [49,52].

(A9) $$K_{FeO/Fe_{2}O_{3}}\left(T\right)=K_{\mathrm{ref}}\exp\left[-\frac{\Delta H_{\mathrm{ref}}}{R}\left(\frac{1}{T}-\frac{1}{T_{\mathrm{ref}}}\right)\right]$$

In Equation (A9), $K_{\mathrm{ref}}=9.94$ at $T_{\mathrm{ref}}=1873\;K$ and $\Delta H_{\mathrm{ref}}=H_{Fe_{2}O_{3}}-2H_{FeO}-0.5H_{O_{2}}$; this equation gives the thermodynamic direction of the ${\mathrm{FeO/Fe}}_{2}O_{3}$ oxidation channel in Table 1.

At a given oxygen potential, the $Fe_{2}O_{3}/FeO$ target ratio is given as described in [49,52].

(A10) $$\Gamma_{Fe^{3+}/Fe^{2+}}=K_{FeO/Fe_{2}O_{3}}\left(T\right)\sqrt{p_{O_{2}}^{slag-gas}}$$

In Equation (A10), $\Gamma_{Fe^{3+}/Fe^{2+}}$ is the target $Y_{Fe_{2}O_{3}}/Y_{FeO}$ ratio; the higher the oxygen potential, the higher the $Fe_{2}O_{3}$ target ratio.

Given the total iron oxide $Y_{\mathrm{FeO},t}$, the target FeO mass fraction is

(A11) $$Y_{FeO}^{eq}=\frac{Y_{\mathrm{FeO},t}}{1+\left(2M_{FeO}/M_{Fe_{2}O_{3}}\right)\Gamma_{Fe^{3+}/Fe^{2+}}}$$

Equation (A11) ensures that the total ${\mathrm{FeO}}_{t}$ is conserved when FeO and ${\mathrm{Fe}}_{2}O_{3}$ interconvert.

The ${\mathrm{Fe}}_{2}O_{3}$ target mass fraction is obtained from the proportional relationship.

(A12) $$Y_{Fe_{2}O_{3}}^{eq}=\Gamma_{Fe^{3+}/Fe^{2+}}Y_{FeO}^{eq}$$

Equations (A12) and (A11) jointly close the gas–slag redox balance.

Total iron oxides are expressed as FeO equivalents. This relation combines FeO and ${\mathrm{Fe}}_{2}O_{3}$ into an ${\mathrm{FeO}}_{t}$ or total-Fe (T.Fe) index for use in the phosphorus-partition relation [2].

(A13) $$Y_{\mathrm{FeO},t}=Y_{FeO}+\frac{2M_{FeO}}{M_{Fe_{2}O_{3}}}Y_{Fe_{2}O_{3}}$$

In Equation (A13), $Y_{\mathrm{FeO},t}$ is the mass fraction of iron oxide converted to FeO.

The T.Fe mass percentage is calculated from the elemental-Fe contents of FeO and ${\mathrm{Fe}}_{2}O_{3}$. It is used in the 2.5log(T.Fe) term of the phosphorus-partition relation [2,81].

(A14) $$T.Fe=100Y_{FeO}\frac{M_{Fe}}{M_{FeO}}+100Y_{Fe_{2}O_{3}}\frac{2M_{Fe}}{M_{Fe_{2}O_{3}}}$$

Equation (A14) shows that T.Fe is not a separate transported species but an instantaneous slag total-iron index calculated from FeO and ${\mathrm{Fe}}_{2}O_{3}$.

Effective basicity places liquid CaO, free CaO, and ${\mathrm{FeO}}_{t}$ on the basic side, and ${\mathrm{SiO}}_{2}$ and $P_{2}O_{5}$ on the acidic side. This relation is used to control CaO solubility and $C_{2}S$–$C_{3}P$ stability [2].

(A15) $$B_{\mathrm{eff}}=\frac{Y_{CaO}+Y_{CaO,free}+Y_{\mathrm{FeO},t}}{Y_{SiO_{2}}+Y_{P_{2}O_{5}}+\varepsilon }$$

In Equation (A15), $B_{\mathrm{eff}}$ is the dimensionless effective basicity used by the present closure, and $\varepsilon =1.0\times 10^{-12}$ avoids a singular value when the acidic inventory approaches zero. The initial input $CaO/SiO_{2}=3.0$ allocates the starting CaO and ${\mathrm{SiO}}_{2}$ inventories and is not identical to the diagnostic $B_{\mathrm{eff}}$.

The slag-to-metal phosphorus partition is given by an Assis–Fruehan-type relation [2,81].

(A16) $$\begin{array}{cc} \log_{10}L_{P}= & 0.073\left[\left(\%CaO\right)+0.8\left(\%P_{2}O_{5}\right)+0.113\left(\%SiO_{2}\right)\right]\\ \; & +2.5\log_{10}\left(T.Fe\right)+\frac{11570}{T}-10.403 \end{array}$$

In Equation (A16), $L_{P}={\left(\%P\right)}_{\mathrm{slag}}/{\left[\%P\right]}_{\mathrm{metal}}$; the parenthesised terms and T.Fe are expressed in mass percent, and *T* is in kelvin. MgO, ${\mathrm{Al}}_{2}O_{3}$, and MnO are not included explicitly, so the equation uses a simplified form consistent with the selected component set.

The capacity of the liquid phase to accommodate CaO is expressed by a bounded empirical equation. This equation represents ${\mathrm{FeO}}_{t}$-assisted CaO entry into the liquid slag, CaO consumption by phosphate, and the inhibition of complete dissolution by the acidic ${\mathrm{SiO}}_{2}$–$P_{2}O_{5}$ network [2,20].

(A17) $$Y_{CaO}^{cap}=\min\left(Y_{\max}^{cap},\max\left(Y_{\min}^{cap},C_{0}+C_{Fe}F_{FeOt}^{CaO}+C_{P}F_{P_{2}O_{5}}^{CaO}-C_{a}F_{a}\right)\right)$$

In Equation (A17), $C_{0}=0.42$, $C_{Fe}=0.20$, $C_{P}=0.08$, $C_{a}=0.15$, $Y_{\min}^{cap}=0.25$, and $Y_{\max}^{cap}=0.70$. Each *F* term is bounded between 0 and 1. These coefficients describe the intended trend and do not replace a multivariate slag phase diagram.

The ${\mathrm{FeO}}_{t}$ solubility factor is calculated as follows:

(A18) $$F_{FeOt}^{CaO}=\frac{Y_{\mathrm{FeO},t}}{Y_{\mathrm{FeO},t}+K_{CaO-FeO}^{liq}}$$

In Equation (A18), $K_{CaO-FeO}^{liq}=0.05$; increasing ${\mathrm{FeO}}_{t}$ increases the CaO capacity of the liquid slag.

The phosphate sink factor is calculated as follows:

(A19) $$F_{P_{2}O_{5}}^{CaO}=\frac{Y_{P_{2}O_{5}}}{Y_{P_{2}O_{5}}+Y_{P_{2}O_{5},1/2}}$$

Equation (A19) increases the fraction of CaO assigned to phosphate-fixation channels as the $P_{2}O_{5}$ inventory increases.

The acidic network penalty factor is calculated as follows:

(A20) $$F_{a}=\frac{Y_{SiO_{2}}+Y_{P_{2}O_{5}}}{Y_{SiO_{2}}+Y_{P_{2}O_{5}}+0.25}$$

Equation (A20) represents inhibition of the complete transfer of free CaO into the liquid phase by the acidic ${\mathrm{SiO}}_{2}$–$P_{2}O_{5}$ network.

The free-CaO equilibrium is given by the excess of total CaO over the liquid-phase capacity [20].

(A21) $$Y_{CaO,free}^{eq}=\max\left(0,Y_{CaO}^{tot}-Y_{CaO}^{cap}\right)$$

Equation (A21) indicates that when the liquid slag cannot accommodate all CaO, the remainder is stored temporarily as free CaO and can re-enter the liquid phase as FeO-assisted dissolution proceeds.

The share of the $C_{3}P$ end member in $C_{2}S$–$C_{3}P$ is determined by the available $P_{2}O_{5}$ [13,15].

(A22) $$\chi_{C3P}=\min\left(1,\max\left(0,\frac{Y_{P_{2}O_{5}}^{prod}}{f_{P_{2}O_{5}}^{C3P}}\frac{Y_{P_{2}O_{5}}^{tot}}{Y_{P_{2}O_{5}}^{tot}+K_{P_{2}O_{5}}}\right)\right)$$

In Equation (A22), $Y_{P_{2}O_{5}}^{prod}=0.12$ and $K_{P_{2}O_{5}}=0.03$. The larger $\chi_{C3P}$ is, the closer the virtual solid phase is to the $3CaO\cdot P_{2}O_{5}$ end member.

The end-member mass fractions follow directly from stoichiometry:

(A23) $$\begin{array}{cccc} f_{CaO}^{C2S} & =\frac{2M_{CaO}}{2M_{CaO}+M_{SiO_{2}}}=0.65116, & f_{SiO_{2}}^{C2S} & =\frac{M_{SiO_{2}}}{2M_{CaO}+M_{SiO_{2}}}=0.34884,\\ f_{CaO}^{C3P} & =\frac{3M_{CaO}}{3M_{CaO}+M_{P_{2}O_{5}}}=0.54237, & f_{P_{2}O_{5}}^{C3P} & =\frac{M_{P_{2}O_{5}}}{3M_{CaO}+M_{P_{2}O_{5}}}=0.45763. \end{array}$$

The values use $M_{CaO}=56.077$, $M_{SiO_{2}}=60.084$, and $M_{P_{2}O_{5}}=141.944\;g\;{\mathrm{mol}}^{-1}$ [95].

The CaO mass fraction in the virtual solid phase is given by a linear mixture of the $C_{2}S$ and $C_{3}P$ end members [15,95].

(A24) $$f_{CaO}^{C2SC3P}=\left(1-\chi_{C3P}\right)f_{CaO}^{C2S}+\chi_{C3P}f_{CaO}^{C3P}$$

Equation (A24) ensures that the mass of CaO consumed in solid-phase generation is consistent with the end-member stoichiometry.

The mass fraction of ${\mathrm{SiO}}_{2}$ in the virtual solid phase comes only from the $C_{2}S$ end member [15,95].

(A25) $$f_{SiO_{2}}^{C2SC3P}=\left(1-\chi_{C3P}\right)f_{SiO_{2}}^{C2S}$$

Equation (A25) shows that as the proportion of $C_{3}P$ increases, the proportion of ${\mathrm{SiO}}_{2}$ in the solid phase decreases.

The mass fraction of $P_{2}O_{5}$ in the virtual solid phase comes only from the $C_{3}P$ end member [15,95].

(A26) $$f_{P_{2}O_{5}}^{C2SC3P}=\chi_{C3P}f_{P_{2}O_{5}}^{C3P}$$

Equation (A26) ensures that phosphorus entering the solid phase is deducted from the liquid-slag $P_{2}O_{5}$ inventory.

The total CaO inventory used in the slag balance includes liquid CaO, free CaO, and CaO bound in the virtual solid phase [15,95].

(A27) $$Y_{CaO}^{tot}=Y_{CaO}+Y_{CaO,free}+f_{CaO}^{C2SC3P}Y_{C2SC3P}$$

Equation (A27) ensures that the total inventory of CaO is conserved when it is transferred between the liquid, free, and solid fixed states.

The total ${\mathrm{SiO}}_{2}$ inventory used in the slag balance includes liquid ${\mathrm{SiO}}_{2}$ and ${\mathrm{SiO}}_{2}$ bound in the virtual solid phase.

(A28) $$Y_{SiO_{2}}^{tot}=Y_{SiO_{2}}+f_{SiO_{2}}^{C2SC3P}Y_{C2SC3P}$$

Equation (A28) closes the ${\mathrm{SiO}}_{2}$ balance during $C_{2}S$ formation and dissolution.

The total $P_{2}O_{5}$ inventory used in the slag balance includes liquid $P_{2}O_{5}$ and $P_{2}O_{5}$ bound in the virtual solid phase.

(A29) $$Y_{P_{2}O_{5}}^{tot}=Y_{P_{2}O_{5}}+f_{P_{2}O_{5}}^{C2SC3P}Y_{C2SC3P}$$

Equation (A29) connects $P_{2}O_{5}$ generated by dephosphorisation with the phosphorus capacity of the liquid slag and the $C_{3}P$ fixation phase.

The $C_{2}S$ saturation index is approximated by the product of CaO and ${\mathrm{SiO}}_{2}$ [15,20].

(A30) $$I_{C2S}=\frac{Y_{CaO}^{2}Y_{SiO_{2}}}{K_{C2S}^{sat}}$$

In Equation (A30), $K_{C2S}^{sat}=2.5\times 10^{-2}$. The index is used to determine whether the local slag composition tends to generate a $C_{2}S$ skeleton.

The $C_{2}S$ saturation factor is represented by a smoothing function [15,23].

(A31) $$F_{sat}={\left[1+\exp\left(-\frac{I_{C2S}-1}{\Delta I_{C2S}}\right)\right]}^{-1}$$

In Equation (A31), $\Delta I_{C2S}=0.05$ is used to avoid step transitions near the saturation point.

The effective-basicity stabilisation factor is given by the following equation:

(A32) $$F_{B}=\frac{B_{\mathrm{eff}}}{B_{\mathrm{eff}}+B_{1/2}}$$

In Equation (A32), $B_{1/2}=2.0$; increasing basicity promotes stabilisation of $C_{2}S$–$C_{3}P$.

The ${\mathrm{FeO}}_{t}$ stability factor is given by the following equation:

(A33) $$F_{\mathrm{FeO},t}=\frac{Y_{\mathrm{FeO},t}}{Y_{\mathrm{FeO},t}+Y_{\mathrm{FeO},t,1/2}}$$

In Equation (A33), $Y_{\mathrm{FeO},t,1/2}=0.25$; this factor represents the role of FeO in promoting lime dissolution and phosphate fixation.

The $P_{2}O_{5}$ stability factor is given by the following equation:

(A34) $$F_{P_{2}O_{5}}=\frac{Y_{P_{2}O_{5}}^{tot}}{Y_{P_{2}O_{5}}^{tot}+Y_{P_{2}O_{5},1/2}}$$

In Equation (A34), $Y_{P_{2}O_{5},1/2}=0.03$; this factor prevents excessive generation of the $C_{3}P$ end member when no phosphate inventory is available.

The phosphorus partitioning stability factor is given by the following equation:

(A35) $$F_{L_{P}}=\frac{L_{P}}{L_{P}+L_{P,1/2}}$$

In Equation (A35), $L_{P,1/2}=100$; a larger slag phosphorus capacity makes solid-phase fixation more likely.

The overall stability factor is obtained by multiplying the five factors.

(A36) $$\begin{array}{cc} F_{\mathrm{stab}}=\min(1,\max(0, & F_{sat}\left(0.35+0.65F_{B}\right)\left(0.50+0.50F_{\mathrm{FeO},t}\right)\\ \; & \times \left(0.35+0.65F_{P_{2}O_{5}}\right)\left(0.50+0.50F_{L_{P}}\right))) \end{array}$$

Equation (A36) shows that the target $C_{2}S$–$C_{3}P$ quantity is constrained simultaneously by silicate saturation, basicity, iron oxide, phosphate, and phosphorus-partition capacity.

The target $C_{2}S$–$C_{3}P$ quantity is determined by the inventory upper limit and the stability factor [15,20,23].

(A37) $$Y_{C2SC3P}^{eq}=F_{\mathrm{stab}}\min\left(Y_{\max}^{C2SC3P},\frac{Y_{CaO}^{tot}}{f_{CaO}^{C2SC3P}},\frac{Y_{SiO_{2}}^{tot}}{f_{SiO_{2}}^{C2SC3P}},\frac{Y_{P_{2}O_{5}}^{tot}}{f_{P_{2}O_{5}}^{C2SC3P}}\right)$$

In Equation (A37), $Y_{\max}^{C2SC3P}=0.65$. The factor $F_{\mathrm{stab}}$ comprises $C_{2}S$ saturation, effective basicity, ${\mathrm{FeO}}_{t}$, $P_{2}O_{5}$, and $L_{P}$; this equation gives the virtual solid-phase target quantity and does not introduce a new fluid phase.

#### Appendix A.1.3. Metal Thermodynamics

The metal phase uses Wagner’s first-order interaction form to estimate the activity coefficient [50].

(A38) $$\log_{10}f_{i}=\sum_{j}e_{i}^{j}\left[\%j\right]$$

In Equation (A38), $e_{i}^{j}$ is the interaction parameter of element *j* on element *i*, and [%j] is the mass percentage of the metal.

For row and column order (C,Si,P,O), the interaction-parameter matrix stored in the UDF is

(A39) $$\left[e_{i}^{j}\right]=\left[\begin{array}{cccc} 0.140 & 0.080 & 0.051 & -0.340\\ 0.180 & 0.110 & 0.090 & -0.230\\ 0.126 & 0.118 & 0.054 & 0.130\\ -0.450 & -0.066 & 0.070 & -0.200 \end{array}\right].$$

The activity of a component in the metal is obtained by multiplying its mass percentage by its activity coefficient [50].

(A40) $$a_{i}=\left[\%i\right]f_{i}$$

In Equation (A40), $a_{C}$, $a_{Si}$, $a_{P}$, and $a_{O}$ are used to calculate the competitive reaction-driving forces of carbon, silicon, phosphorus, and dissolved oxygen. For the silicon equilibrium and rate expressions, the archived UDF approximates silica activity as $a_{SiO_{2}}=\min\left(1,\max\left(0,Y_{SiO_{2}}\right)\right)$.

The carbon–oxygen equilibrium constant is given empirically as described in [50,51].

(A41) $$\log_{10}K_{CO}=\frac{1160}{T}+2.003$$

Equation (A41) corresponds to the reaction [C]+[O]=CO, which is used for the initial dissolved oxygen and carbon–oxygen reaction-driving force.

The silicon–oxygen equilibrium constant is given empirically as described in [50].

(A42) $$\log_{10}K_{Si}=\frac{31,029}{T}-11.912$$

Equation (A42) corresponds to the reaction $\left[Si\right]+2\left[O\right]=SiO_{2}$, so that the competitive priority of silicon for oxygen can change with temperature.

The initial dissolved-oxygen content of the metal is set to the lower of the values given by the carbon–oxygen and silicon–oxygen equilibria [50,51].

(A43) $$Y_{O}^{0}=\frac{1}{100}\min\left(\frac{p_{CO}}{K_{CO}\left[\%C\right]},\sqrt{\frac{a_{SiO_{2}}}{K_{Si}\left[\%Si\right]}}\right)$$

Equation (A43) avoids simultaneously imposing the oxygen contents required by the carbon–oxygen and silicon–oxygen equilibria, thereby reducing spurious transients during initialisation.

### Appendix A.2. Three-Phase Coupled Kinetics

The kinetic closure assigns finite rates and does not treat any equilibrium relation as an instantaneous reaction. This better matches bottom powder injection, where oxygen, CO bubbles, CaO particles, and newly formed FeO films continuously renew the reaction interface in the plume. The top slag region is constrained by practical experience with double-slag operation, hot-metal pretreatment, and high-basicity slag practice [35,67,68,69,70,71,97,98,99,100]. Macroscopic flow-field studies are used to set the scale of bubble agitation, powder trajectories, impact craters, slag entrainment, and mixing times, not to replace the chemical closure [56,57,58,62,72,101,102,103,104].

CaO-particle dissolution is controlled by accessible slag, FeO wetting, and $C_{2}S$ crusting. Hot-metal dephosphorisation and lime-dissolution experiments show that lime does not dissolve completely as soon as it enters the bath. When local ${\mathrm{SiO}}_{2}$ is high or the CaO capacity of the liquid slag is insufficient, a calcium-silicate layer can form on the particle surface and hinder mass transfer. FeO-rich or calcium-ferrite liquid films, in contrast, increase the initial fluxing capacity [5,38,39,40,55,105,106]. The fraction of particle CaO entering liquid slag is therefore written as a bounded function; the remaining CaO first enters the free-CaO inventory and is then converted gradually according to liquid-phase capacity and solid-fixation conditions.

The model does not force gas, slag, and metal into instantaneous global equilibrium. It uses a local reaction framework in which thermodynamics sets the reaction direction, finite kinetics advances the rate, and inventory limits bound the conversion. Reversible reactions first receive forward or reverse directions from equilibrium constants or equilibrium compositions; the actual converted amount is then set by kinetic coefficients, interface area density, and single-step inventory constraints. FeO oxygen release, CO+[O] recombination, $SiO_{2}$ reduction, and rephosphorisation can therefore occur in locally oxygen-poor zones, but small reverse coefficients and single-step limits prevent local equilibrium from being written as an instantaneous complete reaction [23,50].

The normalized driving force for the reversible rate is written as

(A44) $$\Phi_{r}=\frac{\Pi_{r}^{+}-\Pi_{r}^{-}/K_{r}}{\Pi_{r}^{+}+\Pi_{r}^{-}/K_{r}+\varepsilon }$$

In Equation (A44), $\Pi_{r}^{+}$ and $\Pi_{r}^{-}$ are the forward and reverse activity products or partial-pressure products, respectively, and $K_{r}$ is the temperature-dependent equilibrium constant. A positive $\Phi_{r}$ favours the forward reaction, whereas a negative value favours the reverse reaction. Normalisation by the denominator prevents singular source terms near equilibrium.

The oxygen-supply, FeO-capacity, and mixed-contact factors used below are

(A45) $$\begin{array}{cccc} F_{O_{2}} & =\frac{Y_{O_{2}}}{Y_{O_{2}}+0.01}, & F_{FeO} & =\min\left(1,\max\left(0,\frac{0.65-Y_{\mathrm{FeO},t}}{0.65}\right)\right),\\ F_{gm} & =\min(1,\max\left(0,4\alpha_{g}\alpha_{m}\right)), & F_{gs} & =\min(1,\max\left(0,4\alpha_{g}\alpha_{s}\right)),\\ F_{ms} & =I_{ms}\min\left(1,\max\left(0,4\alpha_{m}\alpha_{s}\right)\right). & & \end{array}$$

Here, $I_{ms}=1$ only when both $\alpha_{m}$ and $\alpha_{s}$ are at least 0.01, as in the metal–slag area closure; otherwise, it is zero. The 0.01 and 0.65 values are the oxygen half-saturation mass fraction and the ${\mathrm{FeO}}_{t}$ capacity limit used by the archived UDF. The dissolved-oxygen saturation target and smoothing constant used below are

(A46) $$Y_{O}^{sat}=6.4\times 10^{-4}\sqrt{\max(p_{O_{2}},10^{-12})},\;K_{O}=1.0\times 10^{-4},$$

where $p_{O_{2}}$ is in atm and all *Y* quantities are mass fractions.

The gas–metal FeO-generation channel is controlled by the local oxygen concentration, the gas–metal interfacial area, and the ${\mathrm{FeO}}_{t}$ capacity of the slag [2,5].

(A47) $$r_{FeO}^{form}=k_{FeO}^{form}a_{gm}c_{O_{2}}F_{O_{2}}\left(0.10+0.90F_{FeO}\right)$$

Equation (A47) corresponds to $O_{2}+2Fe\to 2FeO$. Here, $a_{gm}$ is the gas–metal interfacial-area density, $c_{O_{2}}$ is the gas-phase oxygen molar concentration, $F_{O_{2}}$ is the oxygen-supply factor, and $F_{FeO}$ is the ${\mathrm{FeO}}_{t}$ capacity margin. This term represents rapid formation of an FeO-enriched reaction film in the injection zone and is not treated as a universal elementary-reaction constant.

The FeO–metal dissolved-oxygen target used by the forward and reverse driving forces is

(A48) $$\begin{array}{cc} K_{FeO/[O]}\left(T\right) & =0.01\exp\left[-\frac{\Delta H_{FeO\to Fe+[O]}^{ref}}{R}\left(\frac{1}{T}-\frac{1}{1873}\right)\right],\\ Y_{O}^{FeO,eq} & =\min\left(0.005,\max\left(10^{-9},K_{FeO/[O]}\left(T\right)Y_{FeO}\right)\right). \end{array}$$

The reference enthalpy in Equation (A48) is evaluated from the same Fe, dissolved-O, and FeO enthalpy functions used for the reaction-heat source at 1873 K; the reference mass-fraction ratio is 0.01.

The positive driving force for the formation of FeO from metal dissolved oxygen is written as

(A49) $$D_{O\to FeO}=\frac{\max(0,Y_{O}-Y_{O}^{FeO,eq})}{Y_{O}+K_{O}}$$

In Equation (A49), $Y_{O}^{FeO,eq}$ is the dissolved-oxygen mass fraction required for local equilibrium between FeO and the metal. The reaction Fe+[O]→FeO advances only when the metal value $Y_{O}$ exceeds this target.

The reverse driving force for FeO to release oxygen to metal is written as

(A50) $$D_{FeO\to O}=\frac{\max(0,Y_{O}^{FeO,eq}-Y_{O})}{Y_{O}^{FeO,eq}+K_{O}}$$

Equation (A50) indicates that when the metal oxygen content lies below the FeO-equilibrium target, slag FeO returns Fe to the metal and releases [O]. This reverse path retains the oxygen-supply role of FeO in low-oxygen regions.

The forward FeO formation rate at the metal–slag interface is written as

(A51) $$r_{FeO}^{ms}=k_{FeO}^{ms}a_{ms}\frac{\rho_{m}Y_{O}}{M_{O}}D_{O\to FeO}\left(0.10+0.90F_{FeO}\right)$$

Equation (A51) corresponds to Fe+[O]→FeO and, together with Equation (A47), increases ${\mathrm{FeO}}_{t}$ in the slag. This channel is limited by the metal–slag contact area and the dissolved-oxygen inventory of the metal.

The reverse-dissolution and oxygen-release rate of FeO is written as

(A52) $$r_{FeO}^{diss}=k_{FeO}^{diss}a_{ms}\frac{\rho_{s}Y_{FeO}}{M_{FeO}}D_{FeO\to O}$$

Equation (A52) corresponds to FeO→Fe+[O]. The reverse coefficient takes a conservative value below common FeO-reduction mass-transfer coefficients so that ${\mathrm{FeO}}_{t}$ is not depleted instantaneously during the initial oxygen-poor stage [50,59].

The rate at which oxygen directly dissolves into metal is determined by the gas–metal interface, oxygen concentration, and metal oxygen capacity.

(A53) $$r_{O_{2}}^{diss}=k_{O_{2}}^{diss}a_{gm}c_{O_{2}}\frac{\max(0,Y_{O}^{sat}-Y_{O})}{Y_{O}^{sat}+K_{O}}$$

Equation (A53) corresponds to $O_{2}\to 2\left[O\right]$. $Y_{O}^{sat}$ varies with the square root of the local oxygen potential, so the inlet oxygen can either directly form FeO or enter the metal dissolved oxygen reservoir.

Gas-phase CO oxidation adopts a fast forward relaxation channel.

(A54) $$r_{CO}^{ox}=k_{CO}^{ox}\alpha_{g}\frac{\rho_{g}}{M_{CO}}\max\left(0,x_{CO}\sqrt{x_{O_{2}}}-\frac{x_{CO_{2}}}{K_{CO,ox}}\right)$$

Equation (A54) corresponds to $CO+\frac{1}{2}O_{2}\to CO_{2}$ and represents gas-phase post-combustion between decarburisation-generated CO and inlet oxygen. This sub-channel advances only when $O_{2}$ is available and CO has not reached $CO/CO_{2}$ equilibrium; the reverse reduction feedback is carried by Equations (A56) and (A76).

A signed reversible driving force is used between ${\mathrm{CO}}_{2}$ and metal dissolved oxygen.

(A55) $$\Phi_{CO_{2}}=\frac{p_{CO_{2}}-\left(100Y_{O}+\varepsilon \right)p_{CO}/K_{CO_{2},diss}}{p_{CO_{2}}+\left(100Y_{O}+\varepsilon \right)p_{CO}/K_{CO_{2},diss}+\varepsilon }$$

Equation (A55) comes from $CO_{2}=CO+\left[O\right]$. When $\Phi_{CO_{2}}>0$, ${\mathrm{CO}}_{2}$ cracks and supplies oxygen to the metal; when $\Phi_{CO_{2}}<0$, CO+[O] recombines to generate ${\mathrm{CO}}_{2}$.

The net rate of ${\mathrm{CO}}_{2}$ splitting or recombination is written as

(A56) $$r_{CO_{2}}^{diss}=k_{CO_{2}}^{diss}\left(T\right)a_{gm}\Phi_{CO_{2}}$$

Equation (A56) is a signed rate. A positive value represents $CO_{2}\to CO+\left[O\right]$, whereas a negative value represents $CO+\left[O\right]\to CO_{2}$. The forward and reverse directions are limited by the ${\mathrm{CO}}_{2}$ inventory and by the CO and metal–oxygen inventories, respectively [49,51].

The signed driving force for the carbon–oxygen reaction is written as

(A57) $$\Phi_{C}=\frac{a_{C}a_{O}-p_{CO}/K_{CO}}{a_{C}a_{O}+p_{CO}/K_{CO}+\varepsilon }$$

Equation (A57) corresponds to [C]+[O]=CO. Positive values indicate decarburisation, whereas negative values indicate weak recarburisation under a strongly reducing atmosphere; the reverse branch remains small under converter conditions.

The net rate of carbon–oxygen reaction is written as

(A58) $$r_{C}^{ox}=k_{C}\left(T\right)F_{gm}\frac{\rho_{m}Y_{C}}{M_{C}}\Phi_{C}$$

In Equation (A58), positive values consume metal C and O and generate CO, whereas negative values are controlled by the CO inventory and the reverse-reaction limit. Gas-phase CO is not allowed to increase the carbon content of liquid steel spuriously within one time step [51].

The signed driving force for the silicon–oxygen reaction is written as

(A59) $$\Phi_{Si}=\frac{a_{Si}a_{O}^{2}-a_{SiO_{2}}/K_{Si}}{a_{Si}a_{O}^{2}+a_{SiO_{2}}/K_{Si}+\varepsilon }$$

Equation (A59) corresponds to $\left[Si\right]+2\left[O\right]=SiO_{2}$. Positive values indicate silicon oxidation into the slag phase, whereas negative values indicate the tendency of $SiO_{2}$ to reduce back into metal under low-oxygen conditions.

The net rate of silicon–oxygen reaction is written as

(A60) $$r_{Si}^{ox}=k_{Si}\left(T\right)F_{ms}\frac{\rho_{m}Y_{Si}}{M_{Si}}\Phi_{Si}$$

In Equation (A60), the reverse silicon reduction is subject to a more stringent single-step limit than the forward oxidation, so it acts only as thermodynamic feedback in low-oxygen zones and does not dominate the overall process [2,50].

The equilibrium metal-P mass fraction inferred from the phosphorus distribution ratio is

(A61) $$Y_{P}^{eq}=\frac{Y_{P_{2}O_{5}}\left(2M_{P}/M_{P_{2}O_{5}}\right)}{L_{P}}$$

Equation (A61) converts the slag $P_{2}O_{5}$ inventory into an equilibrium concentration comparable with the metal-P concentration. The preceding slag thermodynamic model supplies $L_{P}$, so the dephosphorisation direction depends on slag basicity, ${\mathrm{FeO}}_{t}$, and temperature.

The forward dephosphorisation driving force is written as

(A62) $$D_{P}^{+}=\frac{\max(0,a_{P}/100-Y_{P}^{eq})}{a_{P}/100+\varepsilon }$$

Equation (A62) is positive when the metal-P activity exceeds the slag–metal equilibrium requirement, thereby driving P towards the slag phase.

The reverse phosphorus driving force is written as

(A63) $$D_{P}^{-}=\frac{\max(0,Y_{P}^{eq}-a_{P}/100)}{Y_{P}^{eq}+\varepsilon }$$

Equation (A63) indicates a local rephosphorisation tendency, $P_{2}O_{5}\to 2P+5\left[O\right]$, when the slag-side phosphate inventory is high relative to metal P.

The silicon gate used in the forward kinetic coefficient is

(A64) $$F_{Si}={\left[1+\exp\left(\frac{Y_{Si}-Y_{Si}^{crit}}{\Delta Y_{Si}}\right)\right]}^{-1},\;Y_{Si}^{crit}=1.0\times 10^{-4},\;\Delta Y_{Si}=5.0\times 10^{-5}.$$

It reduces the contact component of the forward coefficient while Si remains above the transition range. The reversible partition relation and the kinetic closure are then coupled in series by defining one effective forward coefficient,

(A65) $$K_{P}^{\mathrm{eff}}=k_{P}\left(T\right)F_{ms}F_{Si}+k_{P}^{tr,+}\left(T\right)a_{ms},$$

where the first term describes unresolved mixed-cell contact and the second introduces the finite metal–slag interfacial-area density [2,81]. Equation (A65) is one composite rate coefficient, not two stoichiometric dephosphorisation reactions. The unbounded candidate rate is therefore written once as

(A66) $$r_{P}^{cand}=K_{P}^{\mathrm{eff}}\frac{\rho_{m}Y_{P}}{M_{P}}D_{P}^{+}.$$

This form makes the serial structure explicit: the slag state determines $L_{P}$ and $D_{P}^{+}$ before the kinetic coefficient can produce a positive candidate. In the UDF, the two terms of $K_{P}^{\mathrm{eff}}$ remain available under the legacy diagnostic names r_p_ox and r_p_transfer_fwd; their sum is exactly Equation (A66). Neither diagnostic is inserted as a species source. The reverse term is evaluated separately through $D_{P}^{-}$ and is not multiplied by the Si gate.

The reverse rephosphorisation rate of phosphorus is written as

(A67) $$r_{P}^{rev}\equiv r_{P}^{tr,-}=k_{P}^{tr,-}\left(T\right)a_{ms}\frac{2\rho_{s}Y_{P_{2}O_{5}}}{M_{P_{2}O_{5}}}D_{P}^{-}$$

Equation (A67) corresponds to the limited reverse reaction $0.5P_{2}O_{5}\to \left[P\right]+2.5\left[O\right]$; $k_{P}^{tr,-}$ is one-tenth of the interfacial-area forward coefficient. The rate is supplied mainly by mobile slag $P_{2}O_{5}$, while only a limited proportion of the phosphorus fixed in the solid solution can be accessed during short-time rephosphorisation [14,82].

#### Appendix A.2.1. Phosphorus Source Assembly

The UDF applies the dephosphorisation controls in series. The slag state first determines $L_{P}$ and, hence, $D_{P}^{+}$ or $D_{P}^{-}$. The positive driving force and the single coefficient in Equation (A65) give the candidate rate in Equation (A66). The available metal-P inventory then limits that candidate:

(A68) $$r_{P}^{P,cap}=\frac{f_{P}^{+}\alpha_{m}\rho_{m}Y_{P}}{M_{P}\Delta t},\;f_{P}^{+}=2.0\times 10^{-2}.$$

(A69) $$r_{P}^{P,lim}=\min\left(1,\frac{r_{P}^{P,cap}}{r_{P}^{cand}+\varepsilon }\right)r_{P}^{cand}.$$

The local oxygen pool available to all forward metal-side reactions is

(A70) $$\begin{array}{cc} r_{O}^{avail}= & \frac{f_{R}^{+}\alpha_{m}\rho_{m}Y_{O}}{M_{O}\Delta t}+2\max\left(0,r_{O_{2}}^{diss}\right)\\ \; & +\max\left(0,r_{FeO}^{diss}\right)+\max\left(0,r_{CO_{2}}^{diss}\right),\;f_{R}^{+}=0.20. \end{array}$$

Carbon oxidation, silicon oxidation, metal-side FeO formation, and phosphorus oxidation compete for Equation (A70). The phosphorus oxygen demand is $q_{P}=2.5r_{P}^{P,lim}$. If the total forward oxygen demand does not exceed $r_{O}^{avail}$, the phosphorus oxygen factor is $f_{P}^{O}=1$. If it does exceed the available pool, the UDF allocates oxygen by the same demand-weighted rule used for the other forward reactions:

(A71) $$\begin{array}{cc} w_{P} & =\max\left(D_{P}^{+},\varepsilon \right)q_{P},\\ r_{O}^{P,alloc} & =r_{O}^{avail}\frac{w_{P}}{w_{FeO}+w_{C}+w_{Si}+w_{P}},\\ f_{P}^{O} & =\min\left(1,\frac{r_{O}^{P,alloc}}{q_{P}+\varepsilon }\right),\;r_{P}^{fwd}=f_{P}^{O}r_{P}^{P,lim}. \end{array}$$

Here, $w_{FeO}$, $w_{C}$, and $w_{Si}$ are the corresponding non-negative weights for metal-side FeO formation, C oxidation, and Si oxidation. Thus, $r_{P}^{fwd}=0$ when $D_{P}^{+}=0$, when no metal P is available, or when no oxygen is allocated to phosphorus oxidation. The contact and area terms have already been combined in $K_{P}^{\mathrm{eff}}$ before the inventory and oxygen limits are applied; they cannot consume separate P or O inventories. The reverse rate is not included in the forward oxygen demand because rephosphorisation releases, rather than consumes, dissolved oxygen. The signed net rate is

(A72) $$r_{P}^{net}=r_{P}^{fwd}-r_{P}^{rev}.$$

The same $r_{P}^{net}$ is used once in each P-containing species source:

(A73) $$\begin{array}{cc} S_{P}^{\left(P\right)} & =-M_{P}r_{P}^{net},\\ S_{O}^{\left(P\right)} & =-2.5M_{O}r_{P}^{net},\\ S_{P_{2}O_{5}}^{\left(P\right)} & =0.5M_{P_{2}O_{5}}r_{P}^{net}. \end{array}$$

The sign of Equation (A72) has a direct physical meaning. If $r_{P}^{net}>0$, metal P and dissolved O are consumed and mobile slag $P_{2}O_{5}$ is generated. If $r_{P}^{net}<0$, mobile $P_{2}O_{5}$ is consumed and P together with dissolved O returns to the metal. If $r_{P}^{net}=0$, this reversible channel produces no P, O, or $P_{2}O_{5}$ source. Subsequent $C_{3}P$ formation consumes mobile $P_{2}O_{5}$ and transfers it into the less accessible $C_{2}S$–$C_{3}P$ inventory. The earlier notation displayed the two terms of $K_{P}^{\mathrm{eff}}$ too much like separate physical reactions. Equations (A61)–(A71) now show their actual serial role: the $L_{P}$ relation fixes the direction; one effective kinetic coefficient fixes the unbounded forward rate; the P inventory and shared oxygen pool limit that rate; and the forward and reverse rates form one signed source. The archived UDF confirms this ordering: the two legacy diagnostic quantities are combined, scaled by the common P cap and the same oxygen-allocation factor, and then assembled as one $r_{P}^{net}$ in the P, O, $P_{2}O_{5}$, and reaction-energy sources.

#### Appendix A.2.2. Gas–Slag Iron-Oxide Exchange

The $FeO/Fe_{2}O_{3}$ relaxation rate controlled by the direct $O_{2}$ oxygen potential is written as

(A74) $$r_{Fe_{2}O_{3}}^{O_{2}}=k_{Fe_{2}O_{3}}\left(T\right)F_{gs}\frac{\rho_{s}}{M_{Fe_{2}O_{3}}}\left(Y_{Fe_{2}O_{3}}^{eq,O_{2}}-Y_{Fe_{2}O_{3}}\right)$$

Equation (A74) is the signed rate. Positive values represent $2FeO+\frac{1}{2}O_{2}\to Fe_{2}O_{3}$; negative values represent $Fe_{2}O_{3}\to 2FeO+\frac{1}{2}O_{2}$.

The gas–slag rate of ${\mathrm{CO}}_{2}$ oxidising FeO is written as

(A75) $$r_{CO_{2}-FeO}=k_{FeOx}^{CO_{2}}\left(T\right)F_{gs}\frac{\rho_{s}}{M_{Fe_{2}O_{3}}}\max\left(0,Y_{Fe_{2}O_{3}}^{eq,CO/CO_{2}}-Y_{Fe_{2}O_{3}}\right)$$

Equation (A75) corresponds to $CO_{2}+2FeO\to CO+Fe_{2}O_{3}$. When the $CO/CO_{2}$ buffer oxygen potential is higher than the iron oxide state in the local slag, this channel pushes FeO toward high-valent iron.

The gas–slag rate at which CO reduces ${\mathrm{Fe}}_{2}O_{3}$ is written as

(A76) $$r_{CO-Fe_{2}O_{3}}=k_{FeOx}^{CO_{2}}\left(T\right)F_{gs}\frac{\rho_{s}}{M_{Fe_{2}O_{3}}}\max\left(0,Y_{Fe_{2}O_{3}}-Y_{Fe_{2}O_{3}}^{eq,CO/CO_{2}}\right)$$

Equation (A76) corresponds to $CO+Fe_{2}O_{3}\to CO_{2}+2FeO$. Thus, CO generated by decarburisation can react with $O_{2}$ in the gas phase or indirectly with high-valent iron in the slag.

The net rate of iron oxide controlled by ${\mathrm{CO/CO}}_{2}$ is

(A77) $$r_{FeOx}^{CO/CO_{2}}=r_{CO_{2}-FeO}-r_{CO-Fe_{2}O_{3}}$$

Equation (A77) connects gas-phase ${\mathrm{CO/CO}}_{2}$ buffering, slag ${\mathrm{FeO/Fe}}_{2}O_{3}$, and metal decarburisation in a closed cycle.

#### Appendix A.2.3. DPM Governing Equations and CaO Mass Transfer

Granular CaO enters the three-phase Eulerian field along the discrete-phase trajectory.

(A78) $$\frac{dx_{p}}{dt}=u_{p}$$

In Equation (A78), $x_{p}$ and $u_{p}$ are the particle position and velocity, respectively.

Particle momentum is governed by the local continuous-phase flow, drag, gravity, and any other enabled interaction forces.

(A79) $$m_{p}\frac{du_{p}}{dt}=F_{D}+F_{g}+F_{other}$$

Equation (A79) shows that the DPM is used to retain powder entry, penetration, residence, escape, and boundary capture. The chemical transformation of powder is not treated as a global uniform CaO source; it must first satisfy the local effective slag-phase contact condition [32,107,108,109].

The stored DPM setup uses the spherical particle-drag law. Brownian motion, Saffman lift, thermophoresis, virtual mass, and the pressure-gradient force are disabled, so these optional contributions are absent from $F_{other}$. The retained mechanical terms are fluid drag and gravity/buoyancy.

The gas–metal contact factor in Equation (A45) is also used in the FeO reaction-film proxy. It approaches 1 when gas and metal each occupy about half of the control volume, and approaches 0 in a single-phase gas or metal cell.

The total FeO production rate is written as

(A80) $$r_{FeO}^{\Sigma }=r_{FeO}^{form}+r_{FeO}^{ms}$$

Equation (A80) combines gas–metal film formation and metal–slag interfacial formation of FeO and is used to determine whether CaO particles encounter an FeO-enriched reaction film.

The equivalent reaction-film phase fraction derived from the FeO production rate is

(A81) $$\alpha_{FeO}^{rate}=0.30F_{gm}\frac{r_{FeO}^{\Sigma }}{r_{FeO}^{\Sigma }+0.02\;\mathrm{mol}\;m^{-3}\;s^{-1}}$$

In Equation (A81), 0.30 is the upper bound on the equivalent slag-phase fraction supplied by the FeO reaction film, and $0.02\;\mathrm{mol}\;m^{-3}\;s^{-1}$ is the half-saturation FeO-production rate.

The effective phase domain through which particle CaO enters the slag is provided by the resolved slag phase, the existing ${\mathrm{FeO}}_{t}$ film, and the newly formed FeO reaction film.

(A82) $$\alpha_{s}^{eff}=\max\left(\alpha_{s},\alpha_{FeO}^{film},\alpha_{FeO}^{rate}\right)$$

In Equation (A82), when $\alpha_{s}\geq 0.02$, the real slag phase is used directly; when $\alpha_{s}\geq 5.0\times 10^{-7}$ and $Y_{\mathrm{FeO},t}\geq 0.10$, the existing ${\mathrm{FeO}}_{t}$ film supplies the minimum effective slag fraction $\alpha_{FeO}^{film}=0.02$; otherwise, only the new reaction film in Equation (A81) can capture CaO particles. CaO is therefore not released in a gas-only or metal-only cell, but it can be captured by a newly formed FeO film before a thick slag layer develops near the inlet. This film is a reaction/contact surrogate used only in the particle-capture and dissolution closure: explicit seeding of a new Eulerian slag volume fraction is disabled in the production UDF.

The FeO wetting factor for dissolving CaO particles is calculated as follows:

(A83) $$W_{CaO}=0.55\frac{Y_{\mathrm{FeO},t}}{Y_{\mathrm{FeO},t}+K_{wet}}+0.25\frac{Y_{CaO}}{Y_{CaO}+0.02}+0.20\frac{Y_{CaO}+Y_{\mathrm{FeO},t}}{Y_{CaO}+Y_{\mathrm{FeO},t}+Y_{SiO_{2}}+Y_{P_{2}O_{5}}+\varepsilon }$$

where $K_{wet}=0.03$. Higher ${\mathrm{FeO}}_{t}$ and liquid-CaO fractions, together with fewer acidic oxides, promote particle-CaO entry into the liquid slag [5,20].

The equivalent dissolution rate of particles after entering the effective slag phase is written as

(A84) $$k_{CaO}^{p}=k_{CaO}\left(T\right)\Lambda_{p}\frac{\alpha_{s}^{eff}}{\alpha_{s}^{min}}\left(0.25+0.75W_{CaO}\right)$$

In Equation (A84), $\Lambda_{p}=1.0$ is the particle-dissolution rate scaling factor, and $\alpha_{s}^{min}=0.02$ is the effective slag-phase threshold. If $\alpha_{s}^{eff}<0.02$, then $k_{CaO}^{p}=0$; the particles move with the gas or continuous phase without releasing CaO to the slag.

The mass-loss fraction in each particle time step is

(A85) $$f_{p}^{loss}=\min\left[0.20,1-\exp(-k_{CaO}^{p}\Delta t_{p})\right]$$

In Equation (A85), 0.20 is the maximum mass fraction that may be lost within one particle substep, and $\Delta t_{p}$ is not less than $10^{-12}\;s$. The exponential form represents first-order dissolution while remaining bounded at both small and large time steps.

The CaO mass released by a particle to the local control volume in one step is

(A86) $$\Delta m_{p}=m_{p}f_{p}^{loss}$$

The mass loss $\Delta m_{p}$ in Equation (A86) occurs only in the effective slag phase or an FeO reaction film. If a particle remains in a gas-only cell or in metal without an FeO film, then $\Delta m_{p}=0$.

The mass-loss flux of the discrete particles is first written to the accumulated CaO source of the control volume.

(A87) $$S_{CaO}^{DPM,*}=\frac{\Delta m_{p}}{\Delta t_{p}V_{c}}$$

In Equation (A87), $V_{c}$ is the control volume. The superscript ∗ indicates that the source is first stored as cumulative particle–continuous-phase exchange. It is read into the slag phase at the next flow-field chemistry update and allocated to the liquid-CaO and free-CaO inventories according to Equation (A92).

The particle mass after dissolution is

(A88) $$m_{p}^{new}=m_{p}-\Delta m_{p}$$

Equation (A88) ensures that the DPM particle mass and continuous-phase CaO input use the same mass-loss amount; no additional particle-phase mass source is introduced, thereby avoiding duplicate counting.

The criterion for complete dissolution of particles is

(A89) $$m_{p}\leq \max\left(10^{-18},10^{-5}m_{p,0}\right)$$

In Equation (A89), $m_{p,0}$ is the initial particle mass. Upon reaching this threshold, the particle mass is set to zero and tracking is terminated to avoid unnecessarily long-lived trajectories with negligible residual mass.

If particles enter the upper gas region, they are removed from the calculation.

(A90) $$y_{p}\geq 1.2\;m$$

Equation (A90) indicates that a particle has left the slag layer and entered the upper gas region; the remaining CaO is therefore not deposited as a slag-phase CaO source.

The deposited mass when the particle hits the boundary is

(A91) $$\Delta m_{p}^{bd}=f_{bd}m_{p}$$

In Equation (A91), $f_{bd}=1.0$ only when impact occurs within an effective slag phase satisfying $\alpha_{s}^{eff}\geq 0.02$, in which case the remaining CaO is deposited into the slag. Contact with a non-slag boundary terminates the particle without depositing CaO. All boundary collisions terminate trajectories, thereby avoiding spurious long-lived residues from repeated particle rebound.

The fraction of CaO entering the liquid state directly is determined by both FeO wetting and the forward FeO-production rate.

(A92) $$F_{direct}=F_{direct}^{max}W_{CaO}\frac{r_{FeO}^{form}+r_{FeO}^{ms}}{r_{FeO}^{form}+r_{FeO}^{ms}+r_{1/2}^{FeO}}$$

In Equation (A92), $F_{direct}^{max}=0.80$ and $r_{1/2}^{FeO}=0.02\;\mathrm{mol}\;m^{-3}\;s^{-1}$. After the particle mass-loss source enters the slag, the fraction $F_{direct}$ is assigned to liquid CaO. The remainder first enters the internal slag inventory as free CaO and subsequently transfers to liquid CaO according to the liquid-phase capacity.

The net relaxation rate between free CaO and liquid CaO is

(A93) $$r_{CaO}^{free}=k_{CaO}^{free}\left(T\right)\frac{\rho_{s}\left(Y_{CaO,free}-Y_{CaO,free}^{eq}\right)}{M_{CaO}}$$

Equation (A93) is a signed rate. A positive value indicates dissolution of free CaO into the liquid slag; a negative value indicates conversion of excess liquid-phase CaO back to free CaO.

The virtual $C_{2}S$–$C_{3}P$ phosphorus-rich phase is controlled by the difference between the local content and the equilibrium target amount.

(A94) $$r_{solid}=\rho_{s}\left[k_{solid}^{+}\max\left(0,Y_{C2SC3P}^{eq}-Y_{C2SC3P}\right)-k_{solid}^{-}\max\left(0,Y_{C2SC3P}-Y_{C2SC3P}^{eq}\right)\right]$$

Equation (A94) allows precipitation and dissolution of the phosphorus-rich phase in both directions. Precipitation fixes $P_{2}O_{5}$, whereas dissolution releases it; however, only a limited fraction of the solid-solution phosphorus inventory is available to the rephosphorisation channel [14,15].

The total solid-phase rate is converted into a $C_{2}S$ reaction rate according to the $C_{2}S$ end-member share.

(A95) $$r_{C2S}=\frac{r_{solid}\left(1-\chi_{C3P}\right)}{M_{SiO_{2}}F_{C2S}}$$

In Equation (A95), $F_{C2S}$ converts the mass of $C_{2}S$ generated per mole of $SiO_{2}$ into the solid-phase mass.

The total solid-phase rate is converted into a $C_{3}P$ reaction rate according to the $C_{3}P$ end-member share.

(A96) $$r_{C3P}=\frac{r_{solid}\chi_{C3P}}{M_{P_{2}O_{5}}F_{C3P}}$$

In Equation (A96), $F_{C3P}$ converts the mass of $C_{3}P$ generated per mole of $P_{2}O_{5}$ into the solid-phase mass.

The $C_{2}S$ mass amplification factor is given by stoichiometry.

(A97) $$F_{C2S}=\frac{2M_{CaO}+M_{SiO_{2}}}{M_{SiO_{2}}}$$

Equation (A97) ensures mass conservation when $2CaO+SiO_{2}$ generates the solid phase.

The $C_{3}P$ mass amplification factor is given by stoichiometry.

(A98) $$F_{C3P}=\frac{3M_{CaO}+M_{P_{2}O_{5}}}{M_{P_{2}O_{5}}}$$

Equation (A98) ensures mass conservation when $3CaO+P_{2}O_{5}$ generates the solid phase.

The three-phase balance is coupled through the local oxygen potential, the dissolved-oxygen pool, and the ${\mathrm{FeO}}_{t}$ inventory. Gas-phase $O_{2}$, the ${\mathrm{CO/CO}}_{2}$ buffer, and slag ${\mathrm{FeO/Fe}}_{2}O_{3}$ provide the oxygen-potential inputs. Oxidation of metal C, Si, and P and formation of FeO compete for the same [O] inventory. If the oxygen supply is insufficient, only the forward oxygen-consuming channels are scaled in proportion to their demands; reverse oxygen-supplying channels are excluded from the forward demand.

(A99) $$\max\left(0,r_{C}^{ox}\right)+2\max\left(0,r_{Si}^{ox}\right)+2.5r_{P}^{fwd}+r_{FeO}^{ms}\leq r_{O}^{avail}$$

Equation (A99) shows that C, Si, and P oxidation and FeO formation are not independent infinite-rate reactions. They compete for the same oxygen pool supplied by $O_{2}$ dissolution, FeO oxygen release, ${\mathrm{CO}}_{2}$ cracking, and the existing dissolved-oxygen inventory. The phosphorus demand contains only the single limited forward flux $r_{P}^{fwd}$ from Equation (A71); the two terms in its effective kinetic coefficient are not counted separately here.

Formation of $C_{2}S$ and $C_{3}P$ is constrained by the total local CaO inventory.

(A100) $$2r_{C2S}+3r_{C3P}\leq \frac{\rho_{s}\left(Y_{CaO}+Y_{CaO,free}\right)}{M_{CaO}\Delta t}$$

Equation (A100) prevents solid-phase formation from consuming more than locally available CaO in one time step.

(A101) $$r_{C3P}\leq \frac{\rho_{s}Y_{P_{2}O_{5}}}{M_{P_{2}O_{5}}\Delta t}+0.5r_{P}^{fwd}$$

Equation (A101) ensures that the phosphorus fixed in $C_{3}P$ can only come from $P_{2}O_{5}$ that already exists or is generated forward in this step.

The phosphorus-recovery rate is limited by the liquid $P_{2}O_{5}$ inventory and the short-term releasable fraction of $C_{2}S$–$C_{3}P$.

(A102) $$r_{P}^{rev}\leq \frac{2f_{P}^{-}\alpha_{s}\rho_{s}\left(Y_{P_{2}O_{5}}+f_{C2SC3P}^{P}Y_{C2SC3P}^{P_{2}O_{5}}\right)}{M_{P_{2}O_{5}}\Delta t}$$

In Equation (A102), $f_{P}^{-}=2.0\times 10^{-3}$ and $f_{C2SC3P}^{P}=0.05$. This constraint allows the phosphorus-rich solid solution to release only a limited fraction of its phosphorus inventory, preventing exaggerated recovery within a single step.

The two FeO-generation pathways share the same slag-phase ${\mathrm{FeO}}_{t}$ capacity.

(A103) $$r_{FeO}^{form}+r_{FeO}^{ms}\leq \frac{f_{FeO}^{+}\alpha_{s}\rho_{s}\max\left(0,Y_{FeO}^{sat}-Y_{\mathrm{FeO},t}\right)}{M_{FeO}\Delta t}$$

Equation (A103) constrains both gas–metal and metal–slag FeO formation. Here, $Y_{FeO}^{sat}=0.65$. The gas–metal and metal–slag pathways are additionally limited by single-step inventory fractions of 0.40 and $2.0\times 10^{-3}$, respectively, and they share a total reactant-inventory limit of 0.20.

The global safeguards applied after the reaction-specific limits are listed in Table A2. They are implementation bounds rather than independently fitted kinetic parameters. Listing them separately avoids conflating a chemical rate coefficient with a numerical cap.

Forward FeO formation, decarburisation, silicon oxidation, and phosphorus oxidation compete for the same dissolved-oxygen reservoir rather than drawing from independent oxygen supplies. The relative demand weights are assigned from the stoichiometric oxygen demand in Table 1: one mole of [C] consumes one mole of [O], one mole of [Si] consumes two moles of [O], and one mole of [P] consumes 2.5 moles of [O] when expressed as $P_{2}O_{5}$ formation [50,81]. The available oxygen pool consists of the consumable dissolved oxygen already present in the metal, oxygen transferred from gas-phase $O_{2}$, oxygen released by FeO reduction, and oxygen supplied by ${\mathrm{CO}}_{2}$ cracking. If the total forward demand exceeds this pool, only the forward oxygen-consuming channels are scaled in proportion to their assigned demand; reverse channels remain limited by their own CO, oxide, phosphate, and metal inventories. This avoids converting a favourable equilibrium target into an unbounded dephosphorisation or desiliconisation rate when local oxygen is insufficient.

**Table A1:** Reversible kinetic parameters and single-step constraints.

Channel	Reference Rate Parameters	Single-Step Constraint	Basis and Purpose
$O_{2}+2Fe\to 2FeO$	$k_{FeO}^{form}=5.0\times 10^{-2}\;m\;s^{-1}$, $E_{a}=32\;\mathrm{kJ}\;{\mathrm{mol}}^{-1}$	The gas–metal FeO-generation single-step inventory fraction is 0.40, and the shared-reactant upper limit is 0.20	Rapid FeO-film generation at the injection hot-spot; requires calibration against the FeO-generation rate [2,5].
Fe+[O]→FeO	$k_{FeO}^{ms}=2.0\times 10^{-4}\;m\;s^{-1}$, $E_{a}=60\;\mathrm{kJ}\;{\mathrm{mol}}^{-1}$	$2.0\times 10^{-3}$	The metal–slag limited mass transfer channel is slower than the injection hot-spot film formation.
FeO→Fe+[O]	$k_{FeO}^{diss}=1.0\times 10^{-5}\;m\;s^{-1}$, $E_{a}=80\;\mathrm{kJ}\;{\mathrm{mol}}^{-1}$	$1.0\times 10^{-4}$	The FeO-reduction mass-transfer coefficient adopts a conservative magnitude to avoid rapid depletion of ${\mathrm{FeO}}_{t}$ [59].
$O_{2}\to 2\left[O\right]$	$k_{O_{2}}^{diss}=2.0\times 10^{-3}\;m\;s^{-1}$, $E_{a}=35\;\mathrm{kJ}\;{\mathrm{mol}}^{-1}$	0.25	Oxygen enters the metal dissolved oxygen reservoir.
$CO+\frac{1}{2}O_{2}\to CO_{2}$	$k_{CO}^{ox}=80\;s^{-1}$, $E_{a}=80\;\mathrm{kJ}\;{\mathrm{mol}}^{-1}$	0.10	Gas-phase afterburning is rapid but limited within each time step [49].
$CO_{2}↔CO+\left[O\right]$	$k_{CO_{2}}^{diss}=12$, $E_{a}=97.3\;\mathrm{kJ}\;{\mathrm{mol}}^{-1}$	Forward decomposition capped at 0.05; reverse limited by CO and O stocks	${\mathrm{CO/CO}}_{2}$–liquid iron C/O balance gives direction [51].
$CO_{2}/CO$–FeOx	$k_{FeOx}^{CO_{2}}=0.02\;s^{-1}$, $E_{a}=80\;\mathrm{kJ}\;{\mathrm{mol}}^{-1}$	Gas-phase redox inventory $5.0\times 10^{-5}$, ${\mathrm{FeO/Fe}}_{2}O_{3}$ shared inventory $5.0\times 10^{-4}$	Gas-phase buffer adjusts the ${\mathrm{FeO/Fe}}_{2}O_{3}$ oxygen potential.
[C]+[O]↔CO	$k_{C}=0.50$, $E_{a}=80\;\mathrm{kJ}\;{\mathrm{mol}}^{-1}$	Forward 0.20, reverse $5.0\times 10^{-3}$	Retain decarburisation and weak recarburisation trends [51].
$\left[Si\right]+2\left[O\right]↔SiO_{2}$	$k_{Si}=0.50$, $E_{a}=90\;\mathrm{kJ}\;{\mathrm{mol}}^{-1}$	Forward 0.20, reverse $1.0\times 10^{-3}$	Silicon is preferentially oxidised during the early stage, and the low-oxygen reverse rate is strongly limited [50].
Composite forward P closure	$k_{P}^{cf}=0.08\;s^{-1}$, $k_{P}^{ia}=2.0\times 10^{-4}\;m\;s^{-1}$, $E_{a}=100\;\mathrm{kJ}\;{\mathrm{mol}}^{-1}$ at $T_{ref}=1873\;K$	One forward P-inventory fraction of 0.02 and one shared dissolved-O allocation are applied after the two coefficient contributions are combined	$L_{P}$ supplies $D_{P}^{+}$; the effective kinetic coefficient, P inventory, and oxygen allocation then control the single forward flux in series [2,82].
Reverse P closure	$k_{P}^{rev}=2.0\times 10^{-5}\;m\;s^{-1}$, $E_{a}=130\;\mathrm{kJ}\;{\mathrm{mol}}^{-1}$	Reverse fraction $2.0\times 10^{-3}$ and solid-solution releasable ratio 0.05	$D_{P}^{-}$ activates rephosphorisation from mobile $P_{2}O_{5}$; the reverse reaction releases P and dissolved O to the metal [14,82].
CaO dissolution and solid-phase fixation	$k_{CaO}=5.0\;s^{-1}$, $k_{CaO}^{free}=0.20\;s^{-1}$, $k_{C2S}=k_{C3P}=0.02$	The effective slag-phase threshold is 0.02, the DPM single-step mass-loss limit is 0.20, the complete-dissolution threshold is $10^{-5}m_{p,0}$, the effective-slag boundary-deposition fraction is 1.0, and no deposition occurs upon top escape	CaO dissolution is controlled by FeO wetting, temperature and the $C_{2}S$ layer [5,15,20].

**Table A2:** Global source-term and composition safeguards in the production UDF.

Quantity	UDF Value	Numerical Role
Effective source-limiting time step	Fluent current time step; fallback $1.0\times 10^{-3}$ s; lower bound $1.0\times 10^{-9}$ s	Prevents division by an invalid or vanishing time increment.
Maximum species-fraction change per step	0.02	Bounds an explicit local species update.
Maximum phase-volume-fraction loss per step	0.001	Prevents a source from exhausting an Eulerian phase in one update.
Absolute phase-mass-source cap	$2.0\times 10^{3}\;\mathrm{kg}\;m^{-3}\;s^{-1}$	Bounds the assembled interphase mass source.
Absolute species-source cap	$5.0\times 10^{2}\;\mathrm{kg}\;m^{-3}\;s^{-1}$	Bounds each phase-owned species source after inventory limiting.
Absolute molar-rate cap	$5.0\times 10^{6}\;\mathrm{mol}\;m^{-3}\;s^{-1}$	Prevents isolated thermodynamic or Arrhenius spikes.
Absolute energy-source cap	$5.0\times 10^{9}\;W\;m^{-3}$	Bounds the summed reaction-enthalpy source.
Minimum metal–slag phase fractions	$\alpha_{m},\alpha_{s}\geq 0.01$	Activates the finite metal–slag contact factor.
Minimum phase fraction for a positive slag-species source	$1.0\times 10^{-3}$	Provides a finite receiving slag inventory for a positive source.
Normalisation caps	Metal minor species 0.20; slag explicit species 0.98; gas reactive species 0.98	Preserves a positive balance-species fraction in each phase.

The shared inventory constraint for the conversion of FeO to $Fe_{2}O_{3}$ is

(A104) $$\max\left(0,r_{Fe_{2}O_{3}}^{O_{2}}\right)+r_{CO_{2}-FeO}\leq \frac{f_{FeO}^{ox}\alpha_{s}\rho_{s}Y_{FeO}}{2M_{FeO}\Delta t_{\mathrm{eff}}}$$

In Equation (A104), the factor 2 follows from the FeO stoichiometric coefficients in $2FeO+\frac{1}{2}O_{2}=Fe_{2}O_{3}$ and $CO_{2}+2FeO=CO+Fe_{2}O_{3}$.

The shared inventory constraint for $Fe_{2}O_{3}$ reduction toward FeO is

(A105) $$\max\left(0,-r_{Fe_{2}O_{3}}^{O_{2}}\right)+r_{CO-Fe_{2}O_{3}}\leq \frac{f_{Fe_{2}O_{3}}^{red}\alpha_{s}\rho_{s}Y_{Fe_{2}O_{3}}}{M_{Fe_{2}O_{3}}\Delta t_{\mathrm{eff}}}$$

Equation (A105) ensures that direct low-oxygen reversion and CO reduction do not consume more than the local $Fe_{2}O_{3}$ inventory in one time step.

### Appendix A.3. Mass-Source Closure

All component mass sources are assembled directly from the stoichiometric channels in Table 1. Gas-phase $O_{2}$, CO, and ${\mathrm{CO}}_{2}$ are updated by oxygen dissolution, CO afterburning, ${\mathrm{CO}}_{2}$ cracking, and the ${\mathrm{CO/CO}}_{2}$-controlled ${\mathrm{FeO/Fe}}_{2}O_{3}$ redox loop. The metal phase is updated by carbon and silicon oxidation, the single signed phosphorus rate, dissolved-oxygen exchange, and the Fe balance associated with FeO formation and FeO reduction. The slag phase is updated by ${\mathrm{FeO/Fe}}_{2}O_{3}$ conversion, $SiO_{2}$ formation and consumption, $P_{2}O_{5}$ generation and recovery, CaO-particle dissolution, free-CaO exchange, and $C_{2}S$–$C_{3}P$ fixation.

This compressed source construction keeps the sign convention simple: positive forward oxidation consumes metal solutes and dissolved oxygen, whereas reverse reduction or rephosphorisation changes the sign of the same channel. The coefficients used in the source terms are the molar masses and stoichiometric factors listed in the reaction table, so the detailed one-line expressions do not need to be repeated for every species. CaO input from DPM particles is first introduced as a local slag inventory source and is then split between liquid CaO and free CaO before being consumed by $C_{2}S$ and $C_{3}P$ formation. This treatment follows the same local-conservation idea used in Eulerian multiphase source-term closures and in kinetic dephosphorisation models with finite slag and metal inventories [23,25,48].

The three-phase total mass closing condition is

(A106) $$S_{g}+S_{s}+S_{m}=0$$

Equation (A106) requires the combined gas-, slag-, and metal-phase mass sources to maintain overall conservation after all reactions and particle inputs are assembled. Numerical limiting changes only the single-step advancement and does not alter the stoichiometric relation above.

### Appendix A.4. Reaction Heat and Energy Source

The total energy source is calculated as described in [25,49] by summing the enthalpy of each reaction multiplied by the corresponding molar rate.

(A107) $$S_{E}=-\sum_{r}\Delta H_{r}\left(T\right)r_{r}$$

In Equation (A107), the exothermic reaction has $\Delta H_{r}<0$ and gives a positive heat source, and the endothermic reaction has $\Delta H_{r}>0$ and gives a negative heat source.

Individual reaction enthalpies are calculated by correcting the standard enthalpies of formation of products and reactants, along with their heat capacities.

(A108) $$\Delta H_{r}\left(T\right)=\sum_{i}\nu_{i}h_{i}\left(T\right)$$

In Equation (A108), $\nu_{i}$ is positive for products and negative for reactants; $h_{i}\left(T\right)$ includes the standard enthalpy of formation and the sensible heat integrated from the reference temperature to the local temperature.

To prevent unphysical temperature jumps from locally large reaction rates, an absolute upper limit is applied to the energy source.

(A109) $$S_{E}^{lim}=\min\left(S_{E}^{max},\max\left(-S_{E}^{max},S_{E}\right)\right)$$

In Equation (A109), $S_{E}^{max}=5.0\times 10^{9}\;W\;m^{-3}$. This constraint only limits the amplitude of heat release in a single step and does not change the thermodynamic sign of each reaction.

## Appendix B. Computational Details

### Appendix B.1. Gas Flow and Initial Fields

The specified bottom-blown gas flow rate is $Q_{g,\mathrm{std}}=100$ standard L $\min^{-1}$, referenced to 298.15K and 1atm. The inlet gas mass flow rate is therefore

(A110) $${\dot{m}}_{g}=\rho_{g,\mathrm{std}}Q_{g,\mathrm{std}}.$$

For the equimolar $O_{2}$–Ar mixture, $\rho_{g,\mathrm{std}}=1.470362\;\mathrm{kg}\;m^{-3}$ at the stated standard reference condition, which gives ${\dot{m}}_{g}=2.45\times 10^{-3}\;\mathrm{kg}\;s^{-1}$. The corresponding local ideal-gas density at 1873K and 1atm is approximately $0.234\;\mathrm{kg}\;m^{-3}$; this hot-state density is reported separately in Table A3 and must not be multiplied by the standard volumetric flow rate.

The initial temperature of the metal, slag, and gas fields is

(A111) $$T^{0}=1873\;K.$$

The subsequent energy source enters through Equation (A107), and the bounded local temperature is returned to the thermodynamic and kinetic closures.

The initial three-layer phase distribution is

(A112) $$\left(\alpha_{m}^{0},\alpha_{s}^{0},\alpha_{g}^{0}\right)=\left\{\begin{array}{cc} (1,0,0), & y<1.0\;m,\\ (0,1,0), & 1.0\;m\leq y<1.2\;m,\\ (0,0,1), & y\geq 1.2\;m. \end{array}\right.$$

The mixed interface develops from this initial field through Eulerian multifluid transport and interphase momentum exchange.

### Appendix B.2. Phase Properties and Interphase Exchange

Table A3 reports the representative fluid-phase properties adopted at the nominal bath temperature of 1873K, together with the constant CaO-particle properties. The liquid-metal density follows critically evaluated liquid-iron data, whereas $\mu_{m}=0.003\;\mathrm{Pa}\;s$ is deliberately selected near the lower end of the experimentally reported range for fully liquid steels [110,111,112]. The slag values represent a fluid, high-basicity steelmaking slag; in particular, 0.10Pas lies within the range measured at 1873 K for a basicity-3 CaO–MgO–SiO_2_–${\mathrm{Al}}_{2}O_{3}$–FeO slag, although that experimental composition is not identical to the simplified slag used here [113,114]. Gas density follows the ideal-gas law, and the listed heat capacity and transport properties are rounded inlet-mixture values for equimolar $O_{2}$–Ar near 1873 K [49,115].

**Table A3:** Representative material properties and dispersed-phase diameters. Fluid-phase thermophysical values correspond to the nominal bath temperature of 1873K and 1 atm unless otherwise stated; density, heat capacity, conductivity, and dynamic viscosity are reported in kg $m^{-3}$, J ${\mathrm{kg}}^{-1}K^{-1}$, W $m^{-1}K^{-1}$, and Pa s, respectively. The gas values refer to the equimolar $O_{2}$–Ar inlet and use ideal-gas density scaling. The CaO-particle properties are constant, and the particles enter at 300K as specified in Table A5.

Material	Density Treatment	Heat-Capacity Treatment	*k*	μ	Diameter
Metal mixture	Constant, 7000	Constant, 820	35	0.003	Continuous primary phase
Slag mixture	Constant, 3000	Constant, 1200	1.5	0.10	3.0 mm
Gas mixture	Ideal gas; 0.234 at the inlet reference state	Mixture value, approximately 810	0.091	$7.9\times 10^{-5}$	2.5 mm
CaO particle	3320	783	2.25	not applicable	100μm

The adopted liquid-metal viscosity is 0.003Pas, and the slag viscosity is 0.10Pas. These values separate the much more viscous slag from the liquid metal and replace the earlier use of a common transport value. The selected metal viscosity should not be interpreted as a universal pure-iron value. The metal heat capacity and conductivity are rounded high-temperature steel/iron inputs; the slag heat capacity and conductivity are representative engineering values within the broad ranges reported for basic oxygen-steelmaking slags [49,112,113,116]. Because slag properties vary strongly with FeO content, basicity, temperature, and solid fraction, the slag entries are model inputs rather than universal constants. Likewise, the gas heat capacity and transport entries describe the inlet mixture rather than the evolving ${\mathrm{CO/CO}}_{2}$-bearing gas everywhere in the domain. The same reference densities are used when local phase inventories are converted into bounded reaction sources. A property-sensitivity study remains necessary before quantitative prediction.

The value $Sc_{t}=0.70$ was retained because it is the documented general Fluent default and no condition-matched turbulent scalar-flux measurement exists for the present gas–slag–metal system [33]. Published reviews also show that $Sc_{t}$ is flow-dependent rather than universal [34]; consequently, this choice documents the present closure but does not establish its quantitative accuracy. Likewise, $D_{i,q}=2.88\times 10^{-5}\;m^{2}\;s^{-1}$ and $D_{\mathrm{floor}}=0$ are implementation inputs, not inferred thermophysical constants.

**Table A4:** Species-transport settings in the archived production case and UDF.

Setting	Archived Value	Interpretation
Molecular diffusivity, $D_{i,q}$	$2.88\times 10^{-5}\;m^{2}\;s^{-1}$ for each Eulerian mixture	Constant-dilute-approximation input retained to reproduce the archived calculation; not independently calibrated for the present melts.
Turbulent Schmidt number, $Sc_{t}$	0.70	Empirical turbulent scalar-flux closure and Fluent default [33]; not a phase material property.
Additional numerical floor, $D_{\mathrm{floor}}$	0	No user-defined diffusion floor is present because the native molecular diffusivity is already positive.
Multicomponent diffusion	disabled	The constant-dilute approximation is used instead of a full Maxwell–Stefan treatment.
Thermal diffusion (Soret effect)	disabled	No temperature-gradient contribution is added to species mass diffusion.
Species enthalpy diffusion in energy equation	enabled	Enthalpy carried by native species diffusion contributes to the energy equation.
Diagnostic storage	70 user-defined-memory locations	Stores rates, sources, and diagnostic fields; it does not replace the native species equations.

The continuum surface-force option is enabled with pairwise surface-tension coefficients of $1.25\;N\;m^{-1}$ for slag–metal, $1.60\;N\;m^{-1}$ for gas–metal, and $0.50\;N\;m^{-1}$ for gas–slag. Native virtual-mass, lift, wall-lubrication, and turbulent-dispersion forces are disabled for the Eulerian phase pairs in the archived case. These switches and coefficients are reported as solver inputs; their adequacy for plume and entrainment prediction has not been established independently.

All Eulerian phase pairs use the Schiller–Naumann drag closure. In compact form,

(A113) $$M_{pq}=K_{pq}\left(u_{p}-u_{q}\right),\;C_{D}=\left\{\begin{array}{cc} \frac{24}{Re}\left(1+0.15Re^{0.687}\right), & Re\leq 1000,\\ 0.44, & Re>1000. \end{array}\right.$$

Here, $K_{pq}$ is the interphase momentum-exchange coefficient, $u_{p}-u_{q}$ is the phase slip velocity, $C_{D}$ is the drag coefficient, and Re is the relative Reynolds number based on the dispersed-phase diameter. Interphase heat transfer uses the Ranz–Marshall correlation:

(A114) $$Nu=2+0.6Re^{1/2}Pr^{1/3},\;h=\frac{Nu\;k_{q}}{d_{p}}.$$

Here, Nu and Pr are the Nusselt and Prandtl numbers, *h* is the interphase heat-transfer coefficient, $k_{q}$ is the continuous-phase thermal conductivity, and $d_{p}$ is the dispersed-phase diameter. The Fluent phase diameters are 2.5 mm for gas and 3.0 mm for dispersed slag. The reaction-area closure uses its own characteristic scales—$d_{gm}=2.5$ mm, $d_{gs}=5.0$ mm and $d_{ms}=20$ mm—because those lengths represent unresolved reactive contact rather than the phase-diameter input alone.

### Appendix B.3. SST Turbulence and DPM Sensible Heat

The *k*–ω SST model used with the Eulerian equations may be written as [117,118]

(A115) $$\frac{\partial (\rho k)}{\partial t}+\nabla \cdot \left(\rho uk\right)=P_{k}-\beta^{*}\rho k\omega +\nabla \cdot \left[(\mu +\sigma_{k}\mu_{t})\nabla k\right],$$

(A116) $$\frac{\partial (\rho \omega )}{\partial t}+\nabla \cdot \left(\rho u\omega \right)=\alpha S^{2}-\beta \rho \omega^{2}+\nabla \cdot \left[(\mu +\sigma_{\omega }\mu_{t})\nabla \omega \right]+2\left(1-F_{1}\right)\rho \sigma_{\omega 2}\frac{\nabla k\cdot \nabla \omega }{\omega },$$

with

(A117) $$\mu_{t}=\frac{\rho a_{1}k}{\max(a_{1}\omega ,SF_{2})}.$$

The standard solver blending functions and coefficients are used; large-eddy simulation (LES) and detached-eddy simulation (DES) are not enabled.

For a CaO parcel, sensible heat follows

(A118) $$m_{p}c_{p,p}\frac{dT_{p}}{dt}=h_{p}A_{p}\left(T_{q}-T_{p}\right),$$

where $h_{p}$ is evaluated with Equation (A114). The Eulerian field acts on particle momentum and temperature. Native particle-to-Eulerian momentum, turbulence, and sensible-heat feedback is disabled in the archived case. Particle dissolution returns only the lost CaO mass through the custom slag-species source, and no second generic DPM mass source is applied.

### Appendix B.4. CaO Particle Injection Settings

**Table A5:** DPM injection settings stored in the supplied case.

Setting	Archived Value
Injection type and location	Surface injection through the central bottom inlet
Particle-size distribution	Monodisperse, $d_{p}=100\;\mu$m; Rosin–Rammler and tabulated distributions disabled
Particle properties and initial temperature	$\rho_{p}=3320\;\mathrm{kg}\;m^{-3}$, $c_{p,p}=783\;J\;{\mathrm{kg}}^{-1}\;K^{-1}$, $k_{p}=2.25\;W\;m^{-1}\;K^{-1}$, $T_{p}^{0}=300\;K$
Injection velocity	$\left(u_{x},u_{y},u_{z}\right)=\left(0,33.3333,0\right)\;m\;s^{-1}$
Mass flow and duration	$9.16666666\times 10^{-2}\;\mathrm{kg}\;s^{-1}$, active from 0 to 10 s
Configured DPM parcels	5000
Dispersion and breakup	Stochastic turbulent dispersion off; secondary-breakup models off
Coupling	One-way momentum and sensible-heat tracking; native continuous-phase feedback off; custom CaO dissolution-mass return on

The monodisperse size is a numerical simplification rather than a measured particle-size distribution. A size-distribution sensitivity test is therefore needed if the model is compared with a specific powder feed.

### Appendix B.5. Initialisation, Inlet Conditions, and Outlet Backflow

The calculation uses two initial molten-steel composition states, denoted Case H and Case L. They have the same initial *P* but different *C*, Si, and dissolved *O*. The slag, gas inlet, CaO feed, outlet backflow, mesh, and numerical limits remain unchanged. Because three metal variables differ, these simulations are not a carbon-only pair. The initial metal vector is specified as $\left(Y_{C}^{0},Y_{Si}^{0},Y_{P}^{0}\right)$, and the initial dissolved oxygen is obtained from the local *C*–*O* and Si–*O* balance described above. The common initial slag is a high-basicity oxidising slag with $CaO/SiO_{2}=3.0$ and $Y_{\mathrm{FeO},t}^{tot}=0.20$. This gives $Y_{SiO_{2}}^{tot}=0.20$ and $Y_{CaO}^{tot}=0.60$ before the liquid-CaO/free-CaO split. The explicit slag mass-fraction vector for $\left(CaO,SiO_{2},FeO,Fe_{2}O_{3},P_{2}O_{5},CaO_{free},C_{2}S--C_{3}P\right)$ is (0.513333,0.200000,0.200000,0,0,0.086667,0). A basicity near 3 and FeO contents around 20 wt.% are typical of oxidising BOF or hot-metal dephosphorisation slags used to promote phosphorus partitioning while retaining enough iron oxide oxygen-supply capacity [1,2,81].

The inlet gas uses $x_{O_{2}}^{in}=0.50$ and $x_{Ar}^{in}=0.50$, with no inlet CO or ${\mathrm{CO}}_{2}$. Argon is retained as an inert carrier so that the oxygen effect can be separated from nitrogen chemistry and from furnace off-gas recirculation. The common CaO input is ${\dot{m}}_{CaO}^{in}=9.166\times 10^{-2}\;\mathrm{kg}\;s^{-1}$, corresponding to a powder-to-gas ratio of 55kg per standard $m^{3}$ at $Q_{g,\mathrm{std}}=1.666\times 10^{-3}$ standard $m^{3}s^{-1}$. The powder rate and DPM treatment follow bottom-blown lime and limestone-powder injection studies in which particle residence, local slag contact, and FeO-assisted dissolution determine the effective CaO utilisation, rather than the nominal feed rate alone [38,39,40].

The pressure-outlet backflow is fixed as a gas-only CO–$CO_{2}$-dominant atmosphere, with $x_{O_{2}}^{bf}=0.001$, $x_{CO}^{bf}=0.600$, $x_{CO_{2}}^{bf}=0.150$, and $x_{Ar}^{bf}=0.249$. The corresponding mass fractions are $Y_{O_{2}}=9.584103\times 10^{-4}$, $Y_{CO}=0.5033784$, $Y_{CO_{2}}=0.1977272$, and $Y_{Ar}=0.2979360$. Only gas is allowed to backflow at the pressure outlet; slag and metal reference values are boundary placeholders with zero volume fraction. This setting supplies a reducing–weakly oxidising top-gas background while leaving CO and ${\mathrm{CO}}_{2}$ inside the bath to be generated by decarburisation, afterburning, ${\mathrm{CO}}_{2}$ cracking, and ${\mathrm{FeO/Fe}}_{2}O_{3}$ gas–slag redox [49,51,52].

**Table A6:** Final case settings and their physical purpose.

Case	Initial Molten-Steel Mass Fractions	Initial Dissolved Oxygen	Purpose
Case H	$Y_{C}^{0}=0.035$, $Y_{Si}^{0}=0.0010$, $Y_{P}^{0}=0.0010$	$Y_{O}^{0}=5.117556\times 10^{-6}$	Composition state with high C, higher Si, and lower dissolved O; used to examine a decarburisation-dominant response of the coupled closure.
Case L	$Y_{C}^{0}=0.001$, $Y_{Si}^{0}=0.0005$, $Y_{P}^{0}=0.0010$	$Y_{O}^{0}=1.052567\times 10^{-4}$	Composition state with low C, lower Si, and higher dissolved O; used to examine the response when more oxygen potential remains available near the slag–metal interface.

The two cases in Table A6 use the same initial slag state and boundary conditions. The common slag conditions are $Y_{CaO}^{tot}=0.60$, $Y_{SiO_{2}}^{tot}=0.20$, $Y_{\mathrm{FeO},t}^{tot}=0.20$, and $Y_{P_{2}O_{5}}^{tot}=0$; the explicit slag compositions are $Y_{CaO}=0.513333$, $Y_{SiO_{2}}=0.20$, $Y_{FeO}=0.20$, $Y_{Fe_{2}O_{3}}=0$, $Y_{CaO,free}=0.086667$, and $Y_{C2SC3P}=0$. The common inlet-gas mole fractions are $x_{O_{2}}^{in}=0.50$ and $x_{Ar}^{in}=0.50$, corresponding to inlet mass fractions $Y_{O_{2}}=0.444750$ and $Y_{Ar}=0.555250$. The common CaO input is ${\dot{m}}_{CaO}^{in}=9.166\times 10^{-2}\;\mathrm{kg}\;s^{-1}$. The common pressure-outlet backflow mole fractions are $x_{O_{2}}^{bf}=0.001$, $x_{CO}^{bf}=0.600$, $x_{CO_{2}}^{bf}=0.150$, and $x_{Ar}^{bf}=0.249$; only gas-phase backflow is permitted.

Accordingly, differences between Case H and Case L are reported as a combined composition-state response. A separate controlled pair with identical Si and dissolved *O* is required before any difference is assigned quantitatively to carbon.

### Appendix B.6. Flow Controls and Solver Settings

The multiphase flow is solved with an Euler–Euler–DPM coupling scheme. The bottom-blown oxygen–lime-powder case is not a two-phase system with one clear free interface. It contains a bubble plume, molten metal, a continuous mixed-slag phase, locally generated FeO films, and dissolving CaO particles. After oxygen enters the bath, iron oxide forms through gas–metal and gas–slag reactions. When CaO particles reach an effective slag region or an FeO-enriched reaction film, they dissolve and replenish the slag CaO inventory. These new slag regions and bubbles are spatially dispersed and often have low local volume fractions. A VOF calculation would have to resolve many low-volume-fraction slag droplets, bubble interfaces, and newly generated slag regions on a limited grid. For the industrial bottom-blowing process considered here, such interface-by-interface resolution would greatly increase grid and time-step requirements and would make stable representation of phase volume fraction, reaction area, and dispersed-phase volume source terms difficult. The Euler–Euler multifluid method is therefore used to describe molten steel, mixed slag, and gas as interpenetrating continua, with phase volume fractions, phase velocities, and phase-owned species as mean-field variables. The DPM retains particle entry, migration, residence, and dissolution. This combination avoids resolving each slag droplet and bubble while keeping oxygen supply, FeO generation, CaO entry into slag, and slag–metal dephosphorisation in the same local conservation framework [23,24,25,26].

The current case is a three-dimensional, double-precision, unsteady multifluid calculation. A pressure-based finite-volume solver is used because molten steel, slag, and gas remain in the low-Mach-number metallurgical-flow range; the dominant variables are phase volume fraction, pressure–velocity coupling, interphase momentum exchange, buoyancy, and reaction source terms, rather than compressible-wave propagation [53]. This choice is also consistent with the pressure-based multiphase implementation used for incompressible and weakly compressible Fluent calculations [33]. The calculation is advanced in time because particle residence, gas-plume growth, FeO accumulation, heat release, and dephosphorisation sources are all history-dependent. Double precision is retained because low-concentration species such as [P], [O], $P_{2}O_{5}$, and free CaO enter the local reaction-window diagnosis.

Pressure–velocity coupling adopts the Phase-Coupled Semi-Implicit Method for Pressure-Linked Equations (SIMPLE) scheme. SIMPLE-type pressure correction is the standard basis for segregated incompressible finite-volume flow solvers [53]. In an Euler–Euler bath containing gas, slag, and metal simultaneously, the phase-coupled form corrects the shared pressure field and interphase momentum response together. This treatment is more robust near the bottom plume, where the phase fraction, pressure gradient, and slip velocity change rapidly [33]. The pressure, momentum, and multiphase relaxation factors are set to 0.3, 0.7, and 0.5, respectively. The lower pressure relaxation suppresses oscillations caused by large density ratios and strong local volume sources, whereas the intermediate momentum and phase relaxation factors retain plume and slag-entrainment structures without destabilising the transient update.

The turbulence model adopts the *k*–ω SST model. The SST formulation blends near-wall *k*–ω behaviour with far-field robustness and shear-stress transport, which is why it is commonly used for engineering flows involving wall shear, adverse pressure gradients, and separation [117]. Its industrial use as a robust Reynolds-averaged Navier–Stokes (RANS) closure for complex engineering CFD has also been documented in later SST experience studies [118]. Here, it provides a reproducible mean mixing field for interphase area, particle residence, and local source terms. LES and DES are not used because the target is furnace-scale reaction closure rather than resolving the full turbulent eddy spectrum. The *k* and ω equations use first-order upwind discretisation to improve robustness in strong-source multiphase regions.

The energy equation is enabled in all calculations and solved in conservative form. It provides the temperature field and carries the reaction-heat feedback into the thermodynamic equilibrium, phosphorus distribution, Arrhenius rates, ${\mathrm{FeO/Fe}}_{2}O_{3}$ oxygen potential, and CaO-particle dissolution. The energy equation is discretised with the second-order upwind method, and the temperature relaxation factor is 1.0. Compared with momentum and phase volume fraction, the temperature field is controlled jointly by volumetric heat release and interphase transport. Second-order upwind discretisation reduces numerical smoothing of local hot-spots, allowing reaction heat, gas-phase cooling, and liquid-metal heat-capacity buffering near bottom-blown oxygen to retain clearer spatial gradients. Effective temperatures in thermodynamic and kinetic calculations are bounded above and below to prevent local numerical anomalies from driving equilibrium constants or rate constants outside their physical range.

The reported production time step is $\Delta t=1.0\times 10^{-4}\;s$. This step advances the continuous-phase flow, phase fractions, energy equation, and DPM trajectories together. The nominal output time is 4.0s after 40,000 steps. This short interval captures the early formation of the gas channel, FeO enrichment, CaO entry into slag, and the initial coupled source-term response; it does not establish an endpoint or long-time process state. No time-step-independence result is claimed.

The spatial discretisation is set hierarchically for stability and physical sensitivity. Body-force-weighted pressure interpolation is used because gravity, density difference, and phase-volume-fraction gradients dominate the bath pressure field [33]. Momentum, phase volume fraction, and turbulence transport are advanced with first-order upwind discretisation to prioritise boundedness near the inlet, gas–slag–metal mixing zone, and strong source-term region. The energy equation uses second-order upwind discretisation to reduce numerical smoothing of local reaction-heating gradients, following the usual finite-volume tradeoff between bounded first-order convection and less diffusive higher-order transport [54].

Residual convergence criteria differ by equation type. Continuity, velocity, phase volume fraction, turbulence, and component transport equations use $10^{-3}$, while the energy equation uses $10^{-6}$. The tighter energy residual is used because temperature directly enters equilibrium constants, phosphorus partitioning, reaction rates, and reaction enthalpies. The residual criteria are combined with non-negativity, phase-existence, and inventory limits so that iterative convergence and physical boundedness are checked together, consistent with source-term stabilisation practice in finite-volume multiphase CFD [53].

The volume-of-fluid formulation is not used for the reported fields. It can be suitable for top-blown oxygen cases with a clearer interface, whereas resolving the dispersed gas–slag–metal reaction region in the present bottom-blown powder-injection case would require substantially finer spatial and temporal resolution.

## Appendix C. Two-Grid Qualitative Mesh-Sensitivity Comparison

The production calculation uses 1,020,102 hexahedral control volumes. To examine whether the principal one-dimensional trends depend strongly on that resolution, an additional calculation was performed on a substantially coarser mesh containing approximately 132,000 cells. The production mesh therefore contains about 7.7 times as many cells. The author-supplied comparison uses Case H; the same central vertical sampling line (x=z=0, 0≤y≤1m); the same governing framework, boundary-condition concept, phase diameters, and closure definitions; and the nominal analysis time t=4.0s. This is a deliberately severe coarsening test. Because only two meshes are available, neither an observed order of accuracy nor an asymptotic range of grid convergence is established, and no Richardson extrapolation is performed. It is therefore a qualitative mesh-sensitivity comparison rather than an observed-order grid-convergence-index (GCI) assessment. Formal discretisation-uncertainty reporting normally uses a systematically refined grid family and an observed convergence assessment [119,120].

Figure A1b shows that the broad axial steel/gas partition is retained after coarsening, including the near-inlet steel-rich segment and the subsequent gas-dominant portion of the sampled line. The curves nevertheless show non-negligible pointwise differences around the plume and phase-transition region. The qualitative ordering therefore persists, while the selected phase-fraction profiles remain mesh-sensitive. Because the slag volume fraction is not plotted, this figure does not directly test convergence of the slag distribution or of the narrow slag–metal mixing region. More generally, the one-dimensional comparison does not establish grid-independent interface thickness, local phase-fraction extrema, entrainment rate, or three-dimensional plume topology.

The chemical profiles are more sensitive. Figure A1a preserves the same axial ordering on both grids: $O_{2}$ decreases with height, $CO_{2}$ first rises and then falls, and CO becomes the dominant upper-plume species. The coarse mesh nevertheless shifts the transition region and changes the local maxima and upper-plume levels, most visibly for CO. This behaviour is expected because each thermodynamic drive, finite reaction rate, inventory cap, and interfacial source is evaluated from the phase fractions and compositions of one control volume. Coarsening changes the amounts of gas, slag, and metal represented in a mixed cell and can therefore delay, broaden, or displace the cell-wise reaction decision. Nonlinear phase-presence thresholds and first-order upwind numerical diffusion add further resolution-dependence. The comparison supports the qualitative axial gas-conversion sequence; it does not establish convergence of the P source, integrated phosphorus removal, DPM capture, peak reaction rates, or exact reaction-front locations.

Computational cost is material for this coupled calculation. At the same time step, the production mesh requires about 7.7 times as many control-volume updates as the coarse mesh. For a uniform three-dimensional refinement, halving the characteristic cell length would increase the cell count by approximately a factor of eight; if the stable time step were also halved, the number of cell updates for a fixed physical duration would rise by approximately a factor of sixteen before accounting for additional nonlinear iterations, interphase coupling, and DPM tracking. This cost scaling motivates targeted refinement and explicit quantities of interest, but it does not replace a convergence analysis.

The flow field is likewise interpreted as a Reynolds-averaged mean field. The *k*–ω SST model, representative phase diameters, Schiller–Naumann drag law, simplified bottom inlet, and fixed phase-property inputs do not resolve the full distribution of bubble sizes, breakup and coalescence, transient surface waves, splashing, or all turbulence scales. The large bath-length scale, high liquid density, and injection-driven velocity can produce a high-Reynolds-number flow despite the liquid-metal viscosity, so the physical bath may contain fluctuations that an RANS closure cannot reproduce explicitly [25,117,118]. Refining this Euler–Euler mesh would improve the resolution of mean-field gradients but would not by itself resolve bubble-scale interfaces or turbulent eddies, and it would not test closure/model-form uncertainty. Similarity between the two axial profiles therefore does not validate the turbulence, drag, or reaction closures.

Accordingly, the mesh comparison supports only the persistence of the broad axial steel/gas partition and gas-conversion ordering under substantial coarsening. It does not support absolute accuracy of local chemical sources, mixing-layer thickness, three-dimensional reaction-zone location, or endpoint removal. Quantitative use would require a systematically refined grid family, a reported observed-convergence metric such as GCI, and a separate time-step study. No time-step-independence result is claimed here.
